# Juncaceae Species as Promising Sources of Phenanthrenes: Antiproliferative Compounds from *Juncus maritimus* Lam

**DOI:** 10.3390/molecules26040999

**Published:** 2021-02-13

**Authors:** Norbert Kúsz, Dóra Stefkó, Anita Barta, Annamária Kincses, Nikoletta Szemerédi, Gabriella Spengler, Judit Hohmann, Andrea Vasas

**Affiliations:** 1 Department of Pharmacognosy, University of Szeged, 6720 Szeged, Hungary; kusznorbert@gmail.com (N.K.); stefko.dori@gmail.com (D.S.); bartaanita96@gmail.com (A.B.); hohmann.judit@szte.hu (J.H.); 2 Department of Medical Microbiology and Immunobiology, University of Szeged, Dóm tér 10, 6720 Szeged, Hungary; kincses.annamaria90@gmail.com (A.K.); szemeredi.nikoletta@med.u-szeged.hu (N.S.); spengler.gabriella@med.u-szeged.hu (G.S.); 3 Interdisciplinary Centre of Natural Products, University of Szeged, Eötvös u. 6, 6720 Szeged, Hungary

**Keywords:** *Juncus maritimus*, maritins A–D, phenanthrene dimers, dihydrophenanthrene

## Abstract

Juncaceae family represents an abundant source of phenanthrenes. In continuation of our work aiming at the isolation of biologically active compounds from Juncaceae species, *Juncus maritimus* Lam. was subjected to phytochemical and pharmacological investigations. The isolation process was carried out by using combined extraction and chromatographic methods. The structures of the obtained chemical compounds were elucidated by spectroscopic analysis, including HRESIMS, 1D (^1^H, ^13^C-JMOD), and 2D (^1^H-^1^H-COSY, HSQC, HMBC, NOESY) NMR spectra. Four new [maritins A–D (**1**–**4**)] and seven known phenanthrenes (**5**–**11**) were isolated from the plant, of which two (**4** and **11**) are phenanthrene dimers composed of effusol monomers. Maritin C (**3**) has an unusual 4,5-ethanophenanthrene skeleton most likely produced by biosynthetic incorporation of a vinyl group into a cyclohexadiene ring. Compounds **1**–**11** were tested for their antiproliferative activity on seven human tumor cell lines (HeLa, HTM-26, T-47D, A2780, A2780cis, MCF-7, KCR) and one normal cell line (MRC-5) using the 3-(4,5-dimethylthiazol-2-yl)-2,5-diphenyltetrazolium bromide (MTT) assay. The dimeric phenanthrenes showed strong antiproliferative activity against T-47D cells with IC_50_ values of 9.1 and 6.2 µM, respectively.

## 1. Introduction

Phenanthrenes, a small group of aromatic secondary metabolites, have recently gained considerable attention due to their structural diversity and promising pharmacological properties. To date, various phenanthrene derivatives have been described from plant species belonging to the Annonaceae, Aristolochiaceae, Cannabaceae, Combretaceae, Euphorbiaceae, Dioscoreaceae, Lauraceae, Malpighiaceae, Orchidaceae, Stemonaceae, and Juncaceae families [1]. Phenanthrenes are reported to possess a wide range of biological activities including pronounced cytotoxic, antiproliferative, and apoptosis induction effects [2,3].

*Juncus maritimus* Lam. is a perennial halophyte herb native to coastal salt marshes regularly flooded with seawater. In the Algerian, Moroccan, and Tunisian folk medicines, preparations of the plant have long been used as analgesic, antiseptic, and anti-inflammatory remedies to treat various ailments, such as infections of the urinary and reproductive systems, injuries, wounds, and skin diseases [4]. The rhizomes of *J. maritimus* are also recommended for insomnia [5]. However, only a few studies investigated the phytochemical constituents of this plant. In one study, the dichloromethane partition of methanol extract obtained from the rhizomes of *J. maritimus* exerted strong antiviral activity. Bioactivity-guided fractionation led to the identification of the known phenanthrene, dehydrojuncusol, as a novel inhibitor of hepatitis C (HCV) replication [6]. Dehydrojuncusol interfered with the function of nonstructural protein NS5A, an essential component of the viral life cycle targeted by many antiviral agents in the treatment of HCV [7]. A recent article described the isolation of the known effusol from *J. maritimus,* which showed significant in vitro antifungal activity against the common wheat pathogen *Zymoseptoria tritici* [8]. The dichloromethane leaf extract of the plant displayed enhanced free radical scavenging activity in a ferric reducing antioxidant power assay [9]. These results clearly demonstrate that *J. maritimus* is worthy of further phytochemical analysis.

As part of our ongoing research program, we describe here the isolation and structure determination of four new [maritins A–D (**1**–**4**)] and seven known (**5**–**11**) phenanthrenes from the methanol extract of *J. maritimus*. The antiproliferative activity of the isolated phenanthrenes was investigated on seven human cancer cell lines (HeLa, HTM-26, T-47D, A2780, A2780cis, MCF-7, KCR) and one normal cell line (MRC-5).

## 2. Results

**Figure 1:**
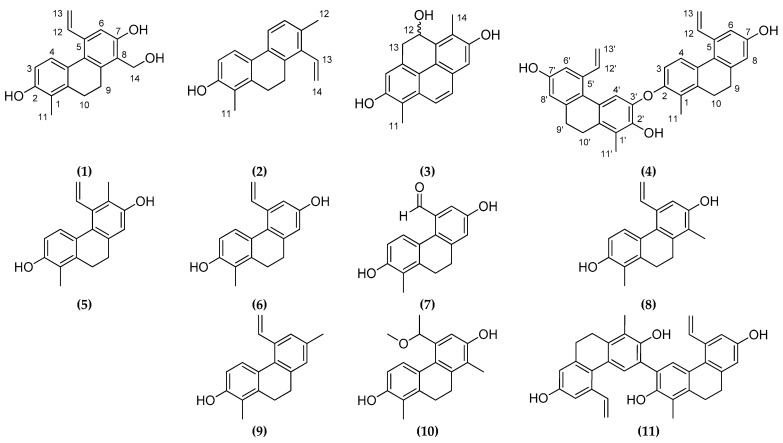
Structures of phenanthrenes (**1**–**11**) isolated from *Juncus maritimus*.

Dried aerial parts of *J. maritimus* were ground and extracted with MeOH at room temperature. After concentration, the extract was dissolved in 50% aqueous MeOH, and solvent–solvent partition was performed with *n*-hexane, chloroform (CHCl_3_), and ethyl acetate (EtOAc). The CHCl_3_ phase was separated and purified with a combination of different chromatographic methods (column chromatography (CC), vacuum liquid chromatography (VLC), medium pressure liquid chromatography (MPLC), preparative thin-layer chromatography (TLC), and HPLC) to afford 11 compounds (Figure 1).

The structure determination was carried out by extensive spectroscopic analysis using 1D (^1^H- and ^13^CJMOD) and 2D (^1^H-^1^H COSY, HSQC, HMBC, and NOESY) NMR and HRMS spectroscopy and comparison of the spectral data with published literature values.

Compound **1** (maritin A) was isolated as a yellow amorphous solid. Its HRESIMS provided the molecular formula C_18_H_18_O_3_ through the presence of a peak at *m/z* 281.1183 [M − H]^−^ (calcd. for C_18_H_17_O_3_, 281.1178). The ^1^H-NMR spectrum displayed signals of two *ortho*-coupled aromatic methines (*δ*_H_ 7.13 d and 6.63 d, *J* = 8.4 Hz), an aromatic proton singlet (*δ*_H_ 6.92), two methylenes (*δ*_H_ 2.76 m and 2.68 m, each 2H), an oxymethylene (*δ*_H_ 4.79 s, 2H), a methyl group (*δ*_H_ 2.21 s, 3H), and a vinyl moiety (*δ*_H_ 6.90 dd, *J* = 17.4 and 10.9 Hz; *δ*_H_ 5.65 dd, *J* = 17.4 and 1.2 Hz; *δ*_H_ 5.18 dd, *J* = 10.9 and 1.2 Hz) (Table 1, Figure S1). The 18 carbon resonances observed in the ^13^C-JMOD NMR spectrum, including two oxygen-bearing sp^2^ carbons at *δ*_C_ 155.1 and 155.3, were attributable to a pentasubstituted phenanthrene derivative.

**Figure 2:**
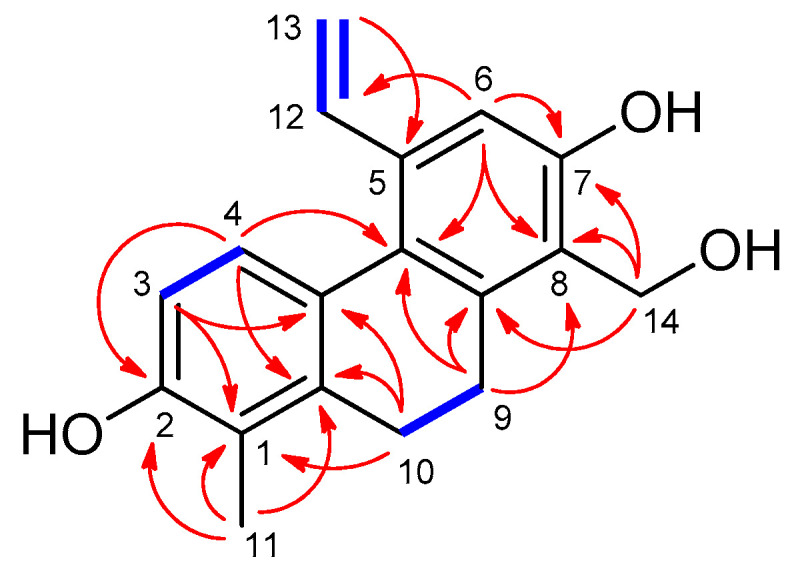
Key HMBC (H→C) and ^1^H-^1^H COSY (**–**) interactions of maritin A (**1**).

The ^1^H-^1^H COSY correlations defined three sequences of correlated protons, namely, –CH_2_–CH_2_– (H_2_-9–H_2_-10), –CH=CH_2_ (H-12–H-13a and H-13b), and –CH=CH– (H-3–H-4) fragments (Figure 2). The structure of compound **1** was assembled with the aid of an HMBC experiment. Heteronuclear long-range correlations of H-3 and H_2_-10 with C-4a (*δ*_C_ 127.2), H-4, H-6, and H_2_-9 with C-5a (*δ*_C_ 128.3), H_2_-9, H_2_-10, and H_2_-14 with C-8a (*δ*_C_ 141.2), as well as of H-4, H_2_-9, H_2_-10, and H_3_-11 with C-1a (*δ*_C_ 140.1) established a 9,10-dihydrophenanthrene skeleton. HMBC correlations from H-3, H-4, and H_3_-11 to C-2 (*δ*_C_ 155.1), and from H-6 and H_2_-14 to C-7 (*δ*_C_ 155.3) suggested that compound **1** contains two hydroxy groups at the positions of C-2 and C-7. The location of the H_3_-11 methyl group at C-1 was dictated by its HMBC correlations with C-1, C-1a, and C-2. The two- and three-bond correlations between H_2_-14 (*δ*_H_ 4.79), C-7, C-8 (*δ*_C_ 124.4), and C-8a demonstrated that the freely rotating hydroxymethyl substituent is attached to C-8. The location of the vinyl moiety at C-5 (*δ*_C_ 136.6) was confirmed by the H-6/C-12 and H-13/C-5 HMBC correlations. The NOE cross-peaks between H-4/H-12, H-13a/H-6, H_2_-9/H_2_-14, and H_2_-10/H_3_-11 were consistent with the proposed structure of **1**, as shown in Figure 2.

Compound **2** (maritin B) was obtained as a white amorphous solid. Its molecular formula was deduced to be C_18_H_18_O based on the protonated molecule in the HRESIMS at *m/z* [M + H]^+^ 251.1429 (calcd. for C_18_H_19_O, 251.1430). The ^1^H-NMR spectrum contained signals of two pairs of *ortho*-coupled aromatic protons (*δ*_H_ 7.47 d and 6.73 d, *J* = 8.2 Hz; 7.49 d and 7.11 d, *J* = 8.4 Hz), two methylenes (*δ*_H_ 2.88 m and 2.74 m, each 2H), a vinyl substituent (*δ*_H_ 6.77 dd, *J* = 17.9 and 11.4 Hz; *δ*_H_ 5.59 dd, *J* = 11.4 and 2.0 Hz; *δ*_H_ 5.22 dd, *J* = 17.9 and 2.0 Hz), and two methyl groups (*δ*_H_ 2.32 s and 2.24 s, each 3H) (Table 1). The HMBC correlations from H_3_-11 (*δ*_H_ 2.24) to C-1 (*δ*_C_ 120.9), C-1a (*δ*_C_ 137.7), and C-2 (*δ*_C_ 153.3), and further correlations between H-3 (*δ*_H_ 6.73), H-4 (*δ*_H_ 7.47), and C-2 showed that a methyl and a hydroxy group are situated on the adjacent carbons C-1 and C-2, respectively. The locations of another methyl (*δ*_H_ 2.32) and a vinyl substituent at C-7 and C-8, respectively, were apparent from the HMBC correlations H_3_-12/C-6, H_3_-12/C-7, H_3_-12/C-8, H-6/C-8, H_2_-9/C-8, and H-14/C-8. Further heteronuclear correlations were detected between H-3, H-5 (*δ*_H_ 7.49), H_2_-10 (*δ*_H_ 2.74), and C-4a (*δ*_C_ 128.4), H-4, H-6 (*δ*_H_ 7.11), H_2_-9 (*δ*_H_ 2.88), and C-5a (*δ*_C_ 133.3), and from H-5, H_2_-9, and H_2_-10 to C-8a (*δ*_C_ 134.1). The NOE cross-peaks H-6/H_3_-12, H_3_-12/H-13, H_2_-9/H-14b, and H_2_-10/H_3_-11 supported the proposed structure of compound **2**.

Separation of the plant extract yielded compound **3** (maritin C) as an orange amorphous solid. According to a peak of the deprotonated molecule at *m/z* 279.1027 [M − H]^−^ in the HRESIMS data, the molecular formula C_18_H_16_O_3_ (calcd. for C_18_H_15_O_3_, 279.1021) was assigned to **3**. The ^1^H-NMR spectrum exhibited two aromatic methines coupled with each other (*δ*_H_ 7.79 and 7.56 d, *J* = 9.2 Hz), two aromatic singlets (*δ*_H_ 7.17 and 7.02), two methyl groups (*δ*_H_ 2.50 s and 2.49 s, each 3H), and signals of an oxymethine (*δ*_H_ 5.45, br s) and a saturated methylene (*δ*_H_ 3.38 and 3.29, each 1H). The ^1^H-^1^H COSY spectrum afforded two structural elements, the aforementioned –CH=CH– (*δ*_H_ 7.79 and 7.56) and a –CH(OR)–CH_2_– fragment (*δ*_H_ 5.45, 3.38, and 3.29). The proton signals at *δ*_H_ 7.02 (H-3) and *δ*_H_ 2.49 (H_3_-11) gave HMBC correlations with a downfield shifted, nonprotonated carbon displayed at *δ*_C_ 153.4, while the aromatic singlet at *δ*_H_ 7.17 (H-8) and the methyl group at *δ*_H_ 2.50 (H_3_-14) gave HMBC correlations to a carbon resonating at *δ*_C_ 155.1. Thus, it was deduced that this phenanthrene bears hydroxy groups at C-2 and C-7. The two methyls were placed onto C-1 and C-6 on the basis of the corresponding H_3_-11/C-1, H_3_-11/C-1a, H_3_-11/C-2, H_3_-14/C-5, H_3_-14/C-6, and H_3_-14/C-7 HMBC correlations. Further long-range correlations from H-9 (*δ*_H_ 7.56) to C-1a, C-5a, and C-8, as well as from H-10 (*δ*_H_ 7.79) to C-1, C-4a, and C-8a established a phenanthrene skeleton with an aromatic ring B. Considering the HMBC cross-peaks of H-13a (*δ*_H_ 3.38) with C-3 (*δ*_C_ 117.0), C-4 (*δ*_C_ 130.9), C-4a (*δ*_C_ 122.5), and C-5 (*δ*_C_ 134.6), it was clear that a vinyl group was incorporated into an oxygen-substituted cyclohexadiene ring. From a biosynthetic point of view, compound **3** was likely formed from a dehydrojuncusol precursor through the modification of its vinylic double bond, followed by a ring closure between C-4 and C-13. The depicted structure of maritin C was corroborated by NOE cross-peaks between H-3/H-13a and b, H-12/H_3_-14, H-8/H-9, and H-10/H_3_-11. The specific optical rotation of **3** was recorded as zero, therefore, it was isolated as a racemic mixture.

Compound **4** (maritin D) has the molecular formula C_34_H_30_O_4_ compatible with its protonated molecule at *m/z* 503.2203 [M + H]^+^ (calcd. for C_34_H_31_O_4_, 503.2222) in the HRESIMS data. The 34 carbon signals displayed in the ^13^C-JMOD NMR spectrum suggested that compound **4** is a phenanthrene dimer (Figure S19). The ^1^H-NMR spectrum, combined with homonuclear ^1^H-^1^H COSY correlations, showed the presence of two vinyl groups (H-12–H_2_-13: *δ*_H_ 6.96 dd, *J* = 17.4 and 10.9 Hz; *δ*_H_ 5.67 d, *J* = 17.4 Hz; *δ*_H_ 5.23 d, *J* = 10.9 Hz; H-12′–H_2_-13′: *δ*_H_ 6.64 dd, *J* = 17.3 and 11.4 Hz; *δ*_H_ 5.33 dd, *J* = 17.3 and 0.9 Hz; *δ*_H_ 4.78 d, *J* = 11.4 Hz), a –CH=CH– (H-3–H-4: *δ*_H_ 7.38 and 6.83 d, *J* = 8.4 Hz) and two –CH_2_–CH_2_– structural portions (H_2_-9–H_2_-10: *δ*_H_ 2.68 m and 2.78 m, each 2H; H_2_-9′–H_2_-10′: *δ*_H_ 2.63 m and 2.64 m, each 2H), and two methyls (H_3_-11: *δ*_H_ 2.28 s; H_3_-11′: *δ*_H_ 2.30 s, each 3H) in **4** (Table 2). 

**Figure 3:**
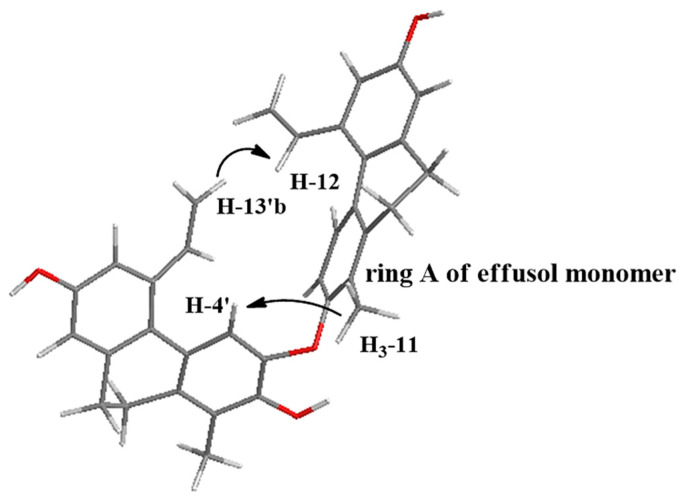
Calculated molecular structure of maritin D (**4**). The arrows indicate diagnostic NOESY correlations. Note that the markedly upfield shifted H-4′ and H-12′–H_2_-13′ are situated in the shielding cone of ring A.

Two pairs of *meta*-coupled aromatic protons (H-6 and H-8: *δ*_H_ 6.88 d and 6.69 d, *J* = 2.2 Hz; H-6′ and H-8′: *δ*_H_ 6.68 br s and 6.61 br s) were also identified via weaker ^4^*J*_H-H_ (*W*-type) COSY cross-peaks and three-bond HMBC correlations between the corresponding methine groups. Further analysis of the HMBC correlations unambiguously determined that **4** is comprised of two monomers of a known 9,10-dihydrophenanthrene, effusol, which was also isolated as an individual compound (**6**) from the plant. Taking into account the HMBC correlations from H-4′ and H_3_-11′ to C-2′ (*δ*_C_ 144.6), it was concluded that oxygen atoms are connected to both of the vicinal carbons C-2′ and C-3′ (*δ*_C_ 156.6). Although no HMBC correlations were observed between the monomers, NOE cross-peaks H-4′/H_3_-11 and H-13′b/H-12 indicated the close proximity of these protons, and consequently implied that the monomers must be attached through an ether bond between C-2/C-2′ or C-2/C-3′. In order to determine the exact structure, energy-minimized structures were generated for each of the hypothetical compounds by using the MM2 force field method. A minimum energy conformation (Figure 3) provided by molecular dynamics calculations was in good agreement with the aforementioned NOE correlations and suggested that the ether bond was formed between C-2/C-3′. The proposed structure was further confirmed by the significantly shielded nature of H-4′ and vinyl resonances H-12′–H_2_-13′ compared to H-4 and H-12–H_2_-13 of the other monomer (Table 2).

This phenomenon was likely caused by the anisotropic effect of aromatic ring A since H-4′ and the vinyl moiety H-12′–H_2_-13′ are located in the shielding cone of ring A. In case of the presence of a C-2/C-2′ linkage, H-4′ and H-12′–H_2_-13′ would be located too far from ring A and, therefore, their chemical shifts would be less affected by the aromatic ring current effects. Considering the above findings, the structure of **4** was formulated as depicted in Figure 1.

Besides the new compounds maritins A–D (**1**–**4**), seven known phenanthrenes, namely, juncusol (**5**) [10], effusol (**6**) [10], 2,7-dihydroxy-1-methyl-5-aldehyde-9,10-dihydrophenanthrene (**7**) [11], 2,7-dihydroxy-1,8-dimethyl-5-vinyl-9,10-dihydrophenanthrene (**8**) [10], juncunol (**9**) [10], jinflexin A (**10**) [12], and effususin A (**11**) [13], were also isolated from *J. maritimus*. Their structures were identified by 1D and 2D NMR spectroscopy, and by comparison of the ^1^H and ^13^C NMR chemical shift values with literature data. All compounds but effusol (**7**) are described for the first time from *J. maritimus*. Moreover, the ^1^H and ^13^C NMR assignments of jinflexin A in methanol-*d*_4_ were not reported previously.

The obtained phenanthrenes **1**–**11** were tested for their antiproliferative activity against seven human tumor cell lines (HeLa, HTM-26, T-47D, A2780, A2780cis, MCF-7, KCR) and one normal human fetal lung fibroblast (MRC-5) cell line using the 3-(4,5-dimethylthiazol-2-yl)-2,5-diphenyltetrazolium bromide (MTT) assay with cisplatin as a positive control (Table 3). Among the tested compounds, dimeric phenanthrenes (**4** and **11**) built up by effusol monomers showed substantial antiproliferative activity against all cell lines investigated. The highest activities were detected on T-47D ductal carcinoma cells (IC_50_ 9.1 μM for **4** and 6.2 μM for **11**, respectively) for both compounds. No significant differences were observed between the effects of dimers on different cell lines.

T-47D cells were the most sensitive to the phenanthrenes, but four compounds (**3**, **4**, **10**, and **11**) displayed remarkable antiproliferative activity (IC_50_ ˂ 20 µM) against A2780cis and MCF-7 cells, too. Maritin B (**2**), juncusol (**5**), and effusol (**6**) were effective only against the HeLa cells (with respective IC_50_ values of 11.0, 0.5, and 2.3 μM). Maritin A (**1**) and compound **8** differ from each other in their substitution at the position of C-8. Based on the experimental data, it seems that replacement of the C-8 methyl group in **8** to a hydroxymethyl moiety present in **1** leads to a drastic increase in the antiproliferative activity. Effusol (**6**) and compound **7** also have similar chemical structures, the difference between them involves the presence of a C-5 vinyl group in **6** and a formyl moiety at the same position in **7**. No significant differences were observed upon comparison of the activities of these phenanthrenes except for the HeLa cell line. Interestingly, effusol (**6**) containing a C-7 hydroxy function displayed much stronger antiproliferative activity on several tumor cell lines than juncunol (**9**), which has a C-7 methyl substituent. This finding suggests that the presence of a polar hydroxy substituent on C-7 is probably more favorable for the antiproliferative effects than its methyl counterpart. Compounds **7**–**9** were weakly effective on all cell lines tested. Although the structure of **8** closely resembles that of jinflexin A (**10**) (the two phenanthrenes contain a vinyl and a methoxyethyl moiety at C-5, respectively), substantial differences were found in their antiproliferative profiles: compound **8** had no effects on any of the cell lines investigated, while **10** exerted activity against all seven tumor cell lines. The presence of a methoxyethyl moiety instead of a vinyl group at C-5 appears to enhance the antiproliferative activity of phenanthrenes. Unfortunately, the tested phenanthrenes showed no selectivity except for compound **1** that exhibited a less antiproliferative effect on MRC-5 normal lung fibroblasts compared to the other cancer cell lines. The other compounds considerably inhibited the proliferation of MRC-5 cells, too.

## 3. Materials and Methods

### 3.1. General Experimental Procedures

Optical rotation was determined in CHCl_3_ at ambient temperature, using a Perkin-Elmer 341 polarimeter (PerkinElmer, MA, USA). NMR spectra were recorded in CDCl_3_, methanol-*d*_4_, and dimethyl sulfoxide-*d*_6_ on a Bruker Avance DRX 500 spectrometer (Bruker, MA, USA) at 500 MHz (^1^H) and 125 MHz (^13^C-JMOD). The signals of the deuterated solvents were taken as references. The chemical shift values (*δ*) were given in ppm and coupling constants (*J*) are expressed in Hz. The high-resolution MS spectra were acquired on a Thermo Scientific Q-Exactive Plus Orbitrap mass spectrometer (Thermo Fisher Scientific, MA USA) equipped with ESI ion source in positive ionization mode. The resolution was over 1 ppm. The data were acquired and processed with MassLynx software (Waters, MA, USA). All solvents used for chromatographic separations and purification steps were analytical or HPLC grade (VWR Ltd., Szeged, Hungary).

For vacuum liquid chromatography (VLC), silica gel (silica gel GF_254_, 15 μm, Merck) and reversed-phase silica (LiChroprep RP-18, 40-63 μm, Merck) were used. Medium-pressure liquid chromatography (MPLC) was performed by a Combi Flash Rf+ Lumen instrument (Teledyne ISCO, NE, USA) on a reversed-phase RediSep Rf HP Gold (50 g) column. Preparative thin-layer chromatography (prep. TLC) was performed on silica gel plates (TLC silica gel 60 F_254_, Merck, Darmstadt, Germany), and on reversed-phase silica gel plates (TLC silica gel 60 RP-18 F_254S_, Merck). Sephadex LH-20 (25–100 μm, Sigma-Aldrich, Budapest, Hungary) was used for gel filtration. HPLC was carried out on a Waters Millipore instrument with UV detection at 254 nm over normal- (Kinetex Luna Silica, 3 μm, 150 × 4.6 mm, Phenomenex Inc., CA, USA) and reversed-phase (Kinetex 5 μm C18, 150 × 4.6 mm and LiChrospher RP-18, 5 μm, 250 × 4 mm) columns.

### 3.2. Plant Material

*Juncus maritimus* Lam. (whole plants, 2.2 kg) was collected in June 2018, near Vir (coordinates: 44°31′80.74” N; 15°05′72.00” E) (Croatia), and identified by one of the authors, László Bakacsy (Department of Plant Biology, University of Szeged, Szeged, Hungary). A voucher specimen (No. 884) has been deposited at the Herbarium of the Department of Pharmacognosy, University of Szeged, Szeged, Hungary.

### 3.3. Extraction and Isolation

The plant material (aerial part) was air-dried (2.2 kg) at room temperature. Thereafter, it was ground and percolated with 40 L methanol at room temperature. After evaporation, the extract was dissolved in 50% aqueous methanol, and repetitive solvent-–solvent partition was performed with 6 × 0.5 L *n*-hexane, 10 × 0.5 L chloroform, and 5 × 0.5 L EtOAc. The concentrated chloroform-soluble fraction (32 g) was separated by vacuum liquid chromatography (VLC) on silica gel with a gradient system of cyclohexane-–EtOAc-–MeOH [from 98:2:0 to 1:1:1 (1500 mL/eluent); volume of collected fractions was 150 mL]. This separation yielded 14 fractions (A-N). The fractions were combined according to their TLC patterns.

All major fractions were purified by Sephadex LH-20 gel chromatography using CH_2_Cl_2_-–MeOH (1:1) as eluent. Fraction B/2 was separated by normal-phase HPLC under gradient conditions, using cyclohexane–EtOAc (19:1 to 9:1 in 10 min and 9:1 to 65:35 in 1 min; flow rate 1.5 mL/min) as mobile phase to obtain compounds **5** (*t*_R_ = 8.3 min, 1.2 mg) and **2** (*t*_R_ = 10.4 min, 2.9 mg). Purification of fractions D/4 and D/5 by preparative TLC afforded compounds **6** (4.3 mg) and **7** (3.4 mg).

Fraction E was chromatographed by reversed-phase MPLC using MeOH–H_2_O (from 8:2 to 1:0). Subfraction E/1 was then further purified by reversed-phase HPLC under gradient conditions, using MeOH–H_2_O (from 45:55 to 82:18 in 10 min; flow rate 1.2 mL/min) as mobile phase to yield compound **10** (*t*_R_ = 5.6 min, 2.4 mg). Subfraction E/2 was separated by preparative TLC on silica gel using cyclohexane–EtOAc–EtOH (20:10:1) as solvent system to yield compounds **9** (3.5 mg) and **3** (4.5 mg).

Fractions H/3 and I/4 were combined (HI/3-4) because of their similar chemical composition and were purified by reversed-phase MPLC using a stepwise gradient solvent system composed of MeOH–H_2_O (from 8:2 to 1:0). Subfraction HI/3-4/1 was separated by preparative TLC on silica gel using cyclohexane–EtOAc–EtOH (20:10:1) as eluent to isolate compound **8** (10.4 mg). HI/3-4/1/2 was purified by reversed-phase HPLC under gradient conditions, using MeOH–H_2_O (from 45:55 to 82:18 in 10 min; flow rate 1.2 mL/min) as mobile phase, and compound **1** (*t*_R_ = 9.0 min, 5.6 mg) was isolated. Subfraction HI/3-4/7 was further fractionated by normal-phase HPLC under gradient conditions, using cyclohexane–EtOAc (from 80:20 to 65:35 in 12 min; flow rate 1.7 mL/min) as mobile phase, to afford compound **11** (*t*_R_ = 13.2 min, 2.3 mg). Subfraction HI/3-4/9 was separated by preparative TLC on silica gel using an isocratic cyclohexane–EtOAc–EtOH (60:30:3) eluent, and then HI/3-4/9/3 was purified by reversed-phase HPLC under gradient conditions, using acetonitrile–H_2_O (from 56:44 to 70:30 in 10 min; flow rate 1.2 mL/min) as mobile phase to yield compound **4** (*t*_R_ = 7.5 min, 2.0 mg).

Maritin A (**1**)

Yellow amorphous solid; for ^1^H- and ^13^C-JMOD NMR (in methanol-*d*_4_) data, see Table 1; HRESIMS *m/z* 281.1183 [M − H]^−^ (calcd. for C_18_H_17_O_3_, 281.1178).

Maritin B (**2**)

White amorphous solid; for ^1^H- and ^13^C-JMOD NMR (in CDCl_3_) data, see Table 1; HRESIMS *m/z* [M + H]^+^ 251.1430 (calcd. for C_18_H_19_O, 251.1430).

Maritin C (**3**)

Orange amorphous solid; [α]^25^_D_ 0 (*c* 0.1, MeOH); for ^1^H-and ^13^C-JMOD NMR (in methanol-*d*_4_) data, see Table 1; HRESIMS *m/z* 279.1027 [M − H]^−^ C_18_H_16_O_3_ (calcd. for C_18_H_15_O_3_, 279.1021).

Maritin D (**4**)

Yellow amorphous solid; for ^1^H- and ^13^C-JMOD NMR (in methanol-*d*_4_) data, see Table 2; HRESIMS *m/z* [M + H]^+^ 503.2203 (calcd. for C_34_H_31_O_4_, 503.2222).

Jinflexin A (**10**):

^1^H-NMR (500 MHz, methanol-*d*_4_): *δ*_H_ 6.67 (1H, d, *J* = 8.3 Hz; H-3), 6.87 (1H, d, *J* = 8.3 Hz; H-4), 6.86 (1H, s; H-6), 2.87 (1H, m; H-9a), 2.37 (1H, m; H-9b), 2.88 (1H, m; H-10a), 2.38 (1H, m; H-10b), 2.22 (3H, s; H_3_-11), 4.85 (1H, overlaps with residual H_2_O signal; H-12), 1.57 (3H, d, *J* = 6.2 Hz; H_3_-13), 2.19 (3H, s; H_3_-14), 2.92 (3H, s; 12-OCH_3_). ^13^C NMR (125 MHz, methanol-*d*_4_): *δ*_C_ 121.8 (C-1), 140.5 (C-1a), 154.9 (C-2), 112.4 (C-3), 127.5 (C-4), 127.4 (C-4a), 129.3 (C-5a), 138.4 (C-5), 111.5 (C-6), 155.1 (C-7), 121.4 (C-8), 139.8 (C-8a), 27.6 (C-9), 26.7 (C-10), 11.8 (C-11), 76.5 (C-12), 23.4 (C-13), 11.8 (C-14), 55.7 (12-OCH_3_).

### 3.4. Antiproliferative Assay

#### 3.4.1. Cell Lines

Breast cancer cell line MCF-7 (ATCC^®®^ HTB-22) and the drug-resistant subline of the human breast cancer MCF-7 (ECACC 86012803; KCR) were purchased from LGC Promochem (Teddington, UK). Both cell lines were cultured in Eagle’s Minimal Essential Medium (EMEM, containing 4.5 g/L glucose) supplemented with a non-essential amino acid mixture, a selection of vitamins, and 10% heat-inactivated fetal bovine serum. In every third passage, 0.56 µg/mL doxorubicin was added to the medium in order to maintain the ABCB1 (P-glycoprotein) expression in KCR cells. A2780 human ovarian cancer cell line (ECACC European Collection of Authentical Cell Culture, Sigma Cat. no. 93112519) and the cisplatin-resistant human ovarian cancer cell line A2780cis (ECACC European Collection of Authentical Cell Culture, Sigma Cat. no. 93112517) were purchased from Merck KGaA (Darmstadt, Germany). The human ovarian cancer cell lines were cultured in RPMI 1640 medium supplemented with 10% heat-inactivated fetal bovine serum. The RPMI 1640 medium of the cisplatin-resistant cell line A2780 was supplemented with 1 µM cisplatin. HeLa (ATCC^®^ CCL-2™) human cervix carcinoma, HTB-26 breast adenocarcinoma, T-47D (ATCC^®^ HTB-133™) ductal carcinoma, and MRC-5 human embryonal lung fibroblast cell lines (ATCC^®^ CCL-171) were purchased from LGC Promochem (Teddington, UK). The cells were cultured in Eagle’s Minimal Essential Medium (EMEM, containing 4.5 g/L glucose) supplemented with a non-essential amino acid mixture, a selection of vitamins, and 10% heat-inactivated fetal bovine serum. HTB-26 cell line was cultured in RPMI 1640 medium supplemented with 10% heat-inactivated fetal bovine serum. T-47D cells were cultured in RPMI 1640 medium supplemented with 10% heat-inactivated fetal bovine serum, 2 mM L-glutamine, 1 mM Na-pyruvate, and 100 mM Hepes. All of the cells were incubated at 37 °C, in a 5% CO_2_, 95% air atmosphere.

#### 3.4.2. Antiproliferative Assay

The antiproliferative effect of the compounds was determined on the human breast (MCF-7, KCR, T-47D, HTB-26), cervical (HeLa), and ovarian (A2780, A2780cis) cancer cells, and on MRC-5 (human embryonic lung fibroblast) cell line. The adherent cells were cultured in 96-well flat-bottomed microtiter plates, using EMEM supplemented with 10% heat-inactivated fetal bovine serum or RPMI 1640 supplemented with 10% heat-inactivated fetal bovine serum, respectively. The density of the cells was adjusted to 6 × 10^3^ cells in 100 μL per well, the cells were seeded for 24 h at 37 °C, 5% CO_2_, then the medium was removed from the plates and fresh medium (100 μL per well) was added to the cells. The effects of increasing concentrations of compounds on cell proliferation were tested in 96-well flat-bottomed microtiter plates. The compounds were diluted in the appropriate medium, the dilutions of compounds were performed in separate plates and then added to the cells. The starting concentration of the compounds was 100 μM, and two-fold serial dilution was performed (concentration range: 100-0.19 μM). The culture plates were incubated at 37 °C for 72 h; at the end of the incubation period, 20 μL of MTT (thiazolyl blue tetrazolium bromide, Sigma) solution (from a stock solution of 5 mg/mL) was added to each well. After incubation at 37 °C for 4 h, 100 μL of sodium dodecyl sulfate (SDS) (Sigma) solution (10% in 0.01 M HCI) were added to each well and the plates were further incubated at 37 °C overnight. Cell growth was determined by measuring the optical density (OD) at 540/630 nm with Multiscan EX ELISA reader (Thermo Labsystems, Cheshire, WA, USA). Mean IC_50_ values were obtained by best fitting the dose-dependent inhibition curves in GraphPadPrism5 program (GraphPad Software version 5.00 for Windows, San Diego, CA, USA) from four parallel experiments for each cell line. Results are expressed in terms of IC_50_, defined as the inhibitory dose that reduces the proliferation of the cells exposed to the tested compounds by 50% [14].

## 4. Conclusions

Eleven phenanthrenes, including four new ones (**1**–**4**), were isolated from the methanolic extract of *J. maritimus*. All compounds but effusol (**7**) were reported for the first time from the plant. Some of the new phenanthrenes possess interesting structural features. The obtained compounds are substituted with hydroxy, methyl, hydroxymethyl, and vinyl groups. In cases of three compounds, the C-5 vinyl groups of biosynthetic intermediates were either incorporated into a cyclohexadiene ring (maritin C, **3**) of a rare 4,5-ethanophenanthrene scaffold or further modified into a formyl (**7**) or a methoxyethyl (**10**) substituent. The new phenanthrene maritin D (**4**) contains two effusol monomers attached to each other through a C-2–C-3′ ether bond, which resulted in the formation of a unique diaryl ether skeleton. The two isolated dimers (**4** and **11**) displayed substantial antiproliferative activity against all investigated cell lines. For both compounds, the highest activities (comparable to the positive control cisplatin) were detected on T-47D ductal carcinoma cells. In general, T-47D cells were the most sensitive to the phenanthrenes, but some of the isolated compounds (e.g., maritin C (**3**) on MCF-7 cells; maritin B (**2**), juncusol (**5**), and effusol (**6**) on HeLa cells) exerted outstanding inhibitory potential against other malignant cell lines, too. An assessment of the results of our pharmacological evaluation allowed us to gain a deeper insight into structure-antiproliferative activity relationships of naturally occurring phenanthrenes.

## Figures and Tables

**Table 1:** ^1^H (500 MHz) and ^13^C (125 MHz) NMR data of compounds **1**–**3**.

Atom	1 ^a^		2 ^b^		3 ^a^	
	*δ*_H_ (*J* in Hz)	*δ*c, Type	*δ*_H_ (*J* in Hz)	*δ*c, Type	*δ*_H_ (*J* in Hz)	*δ*c, Type
1	-	121.6, C	-	120.9, C	-	116.3, C
1a	-	140.1, C	-	137.7, C	-	131.5, C
2	-	155.1, C	-	153.3, C	-	153.4, C
3	6.63, d (8.4)	112.2, CH	6.73, d (8.2)	113.3, CH	7.02, s	117.0, CH
4	7.13, d (8.4)	128.6, CH	7.47, d (8.2)	122.52 *, CH	-	130.9, C
4a	-	127.2, C	-	128.4, C	-	122.47 *, C
5	-	136.6, C	7.49, d (8.4)	122.49 *, CH	-	134.6, C
5a	-	128.3, C	-	133.3, C	-	122.52 *, C
6	6.92, s	113.0, CH	7.11, d (8.4)	128.2, CH	-	125.6, C
7	-	155.3, C	-	134.4, C	-	155.1, C
8	-	124.4, C	-	136.8, C	7.17, s	111.1, CH
8a	-	141.2, C	-	134.1, C	-	130.5, C
9	2.76, m (2H)	26.9, CH_2_	2.88, m (2H)	26.3, CH_2_	7.56, d (9.2)	126.9, CH
10	2.68, m (2H)	26.4, CH_2_	2.74, m (2H)	25.5, CH_2_	7.79, d (9.2)	123.1, CH
11	2.21, s	11.7, CH_3_	2.24, s	11.5, CH_3_	2.49, s	10.7, CH_3_
12	6.90, dd (17.4, 10.9)	140.4, CH	2.32, s	20.8, CH_3_	5.45, br s	67.4, CH
13	5.65, dd (17.4, 1.2) 5.18, dd (10.9, 1.2)	113.2, CH_2_	6.77, dd (17.9, 11.4)	135.1, CH	3.38, br d (16.4) 3.29^+^	38.3, CH_2_
14	4.79, s (2H)	56.6, CH_2_	5.59, dd (11.4, 2.0) 5.22, dd (17.9, 2.0)	120.1, CH_2_	2.50, s	11.4, CH_3_

*^a^* measured in methanol-*d*_4_; *^b^* measured in CDCl_3_; * interchangeable signals; ^+^ overlapped with residual H_2_O signal.

**Table 2:** ^1^H (500 MHz) and ^13^C (125 MHz) NMR data of compound **4** in methanol-*d*_4_.

Effusol Monomer			OH-2 Effusol Monomer		
Atom	*δ*_H_ (*J* in Hz)	*δ*c, Type	Atom	*δ*_H_ (*J* in Hz)	*δ*c, Type
1	-	127.7, C	1′	-	123.4, C
1a	-	140.8, C	1′a	-	133.5, C
2	-	154.4, C	2′	-	144.6, C
3	6.83, d (8.4)	117.8, CH	3′	-	156.6 ^#^, C
4	7.38, d (8.4)	128.7, CH	4′	6.66, s	116.1, CH
4a	-	131.4, C	4′a	-	127.06 *, C
5	-	137.9, C	5′	-	137.4, C
5a	-	126.7, C	5′a	-	126.98 *, C
6	6.88, d (2.2)	113.8^#^, CH	6′	6.68, br s	113.8 ^#^, CH
7	-	157.2, C	7′	-	156.6 ^#^, C
8	6.69 d (2.2)	115.1, CH	8′	6.61, br s	115.0, CH
8a	-	142.1, C	8′a	-	141.7, C
9	2.68, m (2H)	31.4, CH_2_	9′	2.63 ^#^, m (2H)	31.6, CH_2_
10	2.78, m (2H)	26.7, CH_2_	10′	2.64 ^#^, m (2H)	26.1, CH_2_
11	2.28, s	12.4, CH_3_	11′	2.30, s	12.0, CH_3_
12	6.96, dd (17.4, 10.9)	140.1, CH	12′	6.64 dd (17.3, 11.4)	140.2, CH
13	5.67, d (17.4) 5.23, d (10.9)	114.2, CH_2_	13′	5.33, dd (17.3, 0.9) 4.78, d (11.4)	113.7, CH_2_

# overlapping signals; * interchangeable signals.

**Table 3:** Antiproliferative activity (IC_50_) of the tested phenanthrenes **1**–**11**.

Compound	IC_50_ (µM) ± SD							
	HeLa	HTB-26	T-47D	A2780	A2780cis	MCF-7	KCR	MRC-5
1	57.0 ± 1.3	48.7 ± 1.6	12.8 ± 0.9	21.5 ± 0.4	40.1 ± 0.4	34.7 ± 3.0	57.6 ± 0.7	75.4 ± 1.8
2	11.0 ± 0.9	>100	>100	>100	>100	97.0 ± 0.3	>100	>100
3	43.2 ± 0.7	35.9 ± 1.5	17.0 ± 0.6	23.7 ± 0.1	18.6 ± 0.1	9.8 ± 0.6	>100	15.0 ± 0.2
4	22.5 ± 1.2	25.1 ± 0.3	9.1 ± 0.4	14.1 ± 1.3	13.3 ± 0.1	14.2 ± 0.3	16.2 ± 0.6	17.1 ± 3.1
5	0.5 ± 0.0	41.7 ± 3.5	25.0 ± 0.4	23.8 ± 1.3	37.1 ± 2.8	37.1 ± 1.1	35.8 ± 1.7	40.9 ± 2.0
6	2.3 ± 0.7	57.0 ± 2.73	24.6 ± 1.9	33.1 ± 3.1	30.4 ± 0.4	48.6 ± 3.4	39.3 ± 1.6	60.1 ± 5.1
7	24.7 ± 0.5	85.3 ± 4.5	26.6 ± 1.1	30.0 ± 3.6	32.3 ± 2.3	38.0 ± 2.0	70.6 ± 3.1	71.1 ± 1.3
8	>100	>100	57.0 ± 7.1	>100	>100	69.5 ± 1.7	>100	>100
9	76.7 ± 1.8	75.0 ± 5.0	41.4 ± 5.8	43.5 ± 1.1	52.8 ± 2.4	45.7 ± 2.4	68.4 ± 2.5	78.4 ± 3.7
10	24.7 ± 0.3	22.8 ± 0.2	14.2 ± 1.1	22.3 ± 2.7	16.9 ± 4.7	12.9 ± 0.2	24.2 ± 2.1	18.9 ± 4.0
11	25.2 ± 0.6	24.7 ± 2.1	6.2 ± 0.1	25.6 ± 2.4	16.3 ± 0.3	14.0 ± 0. 6	19.6 ± 0.9	20.1 ± 1.6
cisplatin	2.3 ± 0.1	20.1 ± 2.3	5.9 ± 0.1	3.6 ± 0.3	7.3 ± 0.2	0.9 ± 0.0	6.5 ± 0.3	0.6 ± 0.0

## Supplementary Materials

Supplementary materials are available online, Figures S1–S26.

## Sample Availability

Not availability.
