# Supplementary material for: Juncaceae Species as Promising Sources of Phenanthrenes: Antiproliferative Compounds from Juncus maritimus Lam
Source: Molecules. 2021 Feb 13;26(4):999. doi: 10.3390/molecules26040999 (PMC7918049; doi:10.3390/molecules26040999)
Supplement: Supplementary file 1 [file molecules-26-00999-s001.pdf]

Supporting information

Juncaceae Species as Promising Sources of Phenanthrenes: Biologically Active Compounds From *Juncus maritimus* Lam.

Norbert Kúsz [1,†], Dóra Stefkó [1,†], Anita Barta [1], Annamária Kincses [2], Nikoletta Szemerédi [2], Gabriella Spengler [2], Judit Hohmann [1,3], and Andrea Vasas [1,*]

[1] Department of Pharmacognosy, Interdisciplinary Excellence Centre, University of Szeged, 6720 Szeged, Hungary; kusznorbert@gmail.com; stefko.dori@gmail.com; bartaanita96@gmail.com

[2] Department of Medical Microbiology and Immunobiology, University of Szeged, Dóm tér 10, 6720 Szeged, Hungary; kincses.annamaria90@gmail.com; szemeredi.nikoletta@med.u-szeged.hu; spengler.gabriella@med.u-szeged.hu

[3] Interdisciplinary Centre of Natural Products, University of Szeged, Eötvös u. 6, 6720 Szeged, Hungary; hohmann.judit@szte.hu

[†] First authors

* Correspondence: vasasa@pharmacognosy.hu; Tel.: +36-62-546-451

TABLE OF CONTENTS

Figure S1. ^{1}H NMR spectrum (500 MHz) of maritin A (**1**) in methanol-d_4. 3
Figure S2. ^{13}C JMOD NMR spectrum (125 MHz) of maritin A (**1**) in methanol-d_4. 3
Figure S3. HSQC spectrum of maritin A (**1**) in methanol-d_4. 4
Figure S4. ^{1}H-^{1}H COSY spectrum of maritin A (**1**) in methanol-d_4. 4
Figure S5. HMBC spectrum of maritin A (**1**) in methanol-d_4. 5
Figure S6 NOESY spectrum of maritin A (**1**) in methanol-d_4. 5
Figure S7. ^{1}H NMR spectrum (500 MHz) of maritin B (**2**) in $CDCl_3$. 6
Figure S8. ^{13}C JMOD NMR spectrum (125 MHz) of maritin B (**2**) in $CDCl_3$. 6
Figure S9. HSQC spectrum of maritin B (**2**) in $CDCl_3$. 7
Figure S10. ^{1}H-^{1}H COSY spectrum of maritin B (**2**) in $CDCl_3$. 7
Figure S11. HMBC spectrum of maritin B (**2**) in $CDCl_3$. 8
Figure S12. NOESY spectrum of maritin B (**2**) in $CDCl_3$. 8
Figure S13. ^{1}H NMR spectrum (500 MHz) of maritin C (**3**) in methanol-d_4. 9
Figure S14. ^{13}C JMOD NMR spectrum (125 MHz) of maritin C (**3**) in methanol-d_4. 9
Figure S15. HSQC spectrum of maritin C (**3**) in methanol-d_4. 10
Figure S16. ^{1}H-^{1}H COSY spectrum of maritin C (**3**) in methanol-d_4. 10
Figure S17. HMBC spectrum of maritin C (**3**) in methanol-d_4. 11
Figure S18. NOESY spectrum of maritin C (**3**) in methanol-d_4. 11
Figure S19. ^{1}H NMR spectrum (500 MHz) of maritin D (**4**) in methanol-d_4. 12
Figure S20. ^{13}C JMOD NMR spectrum (125 MHz) of maritin D (**4**) in methanol-d_4. 12
Figure S21. HSQC spectrum of maritin D (**4**) in methanol-d_4. 13
Figure S22. ^{1}H-^{1}H COSY spectrum of maritin D (**4**) in methanol-d_4. 13
Figure S23. HMBC spectrum of maritin D (**4**) in methanol-d_4. 14
Figure S24. NOESY spectrum of maritin D (**4**) in methanol-d_4. 14
Figure S25. ^{1}H NMR spectrum (500 MHz) of jinflexin A (**10**) in methanol-d_4. 15
Figure S26. ^{13}C JMOD NMR spectrum (125 MHz) of jinflexin A (**10**) in methanol-d_4. 15

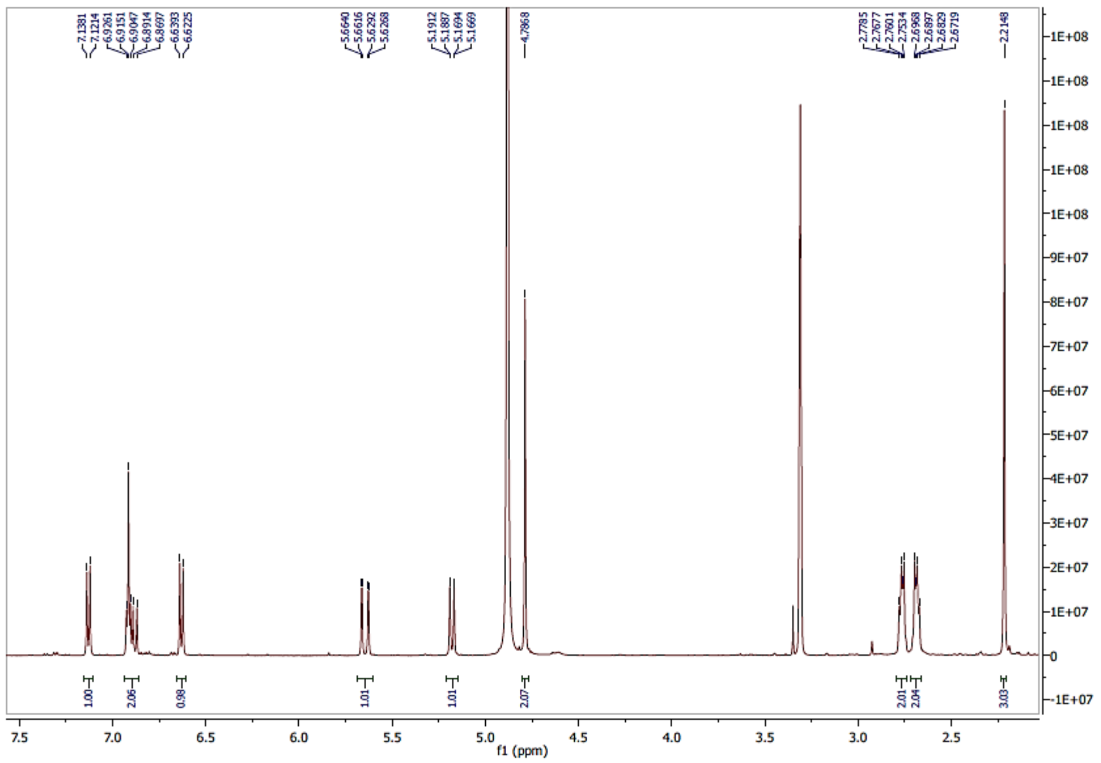

Figure S1. ^{1}H NMR spectrum (500 MHz) of maritin A (**1**) in methanol-d_4.

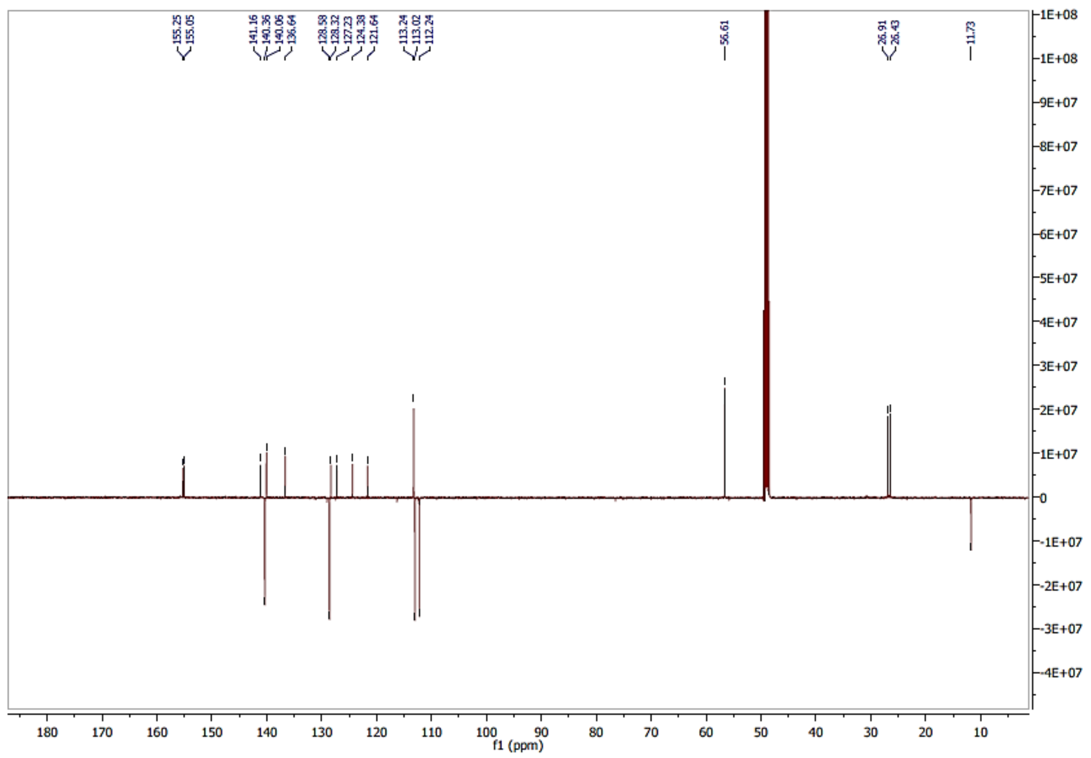

Figure S2. ^{13}C JMOD NMR spectrum (125 MHz) of maritin A (**1**) in methanol-d_4.

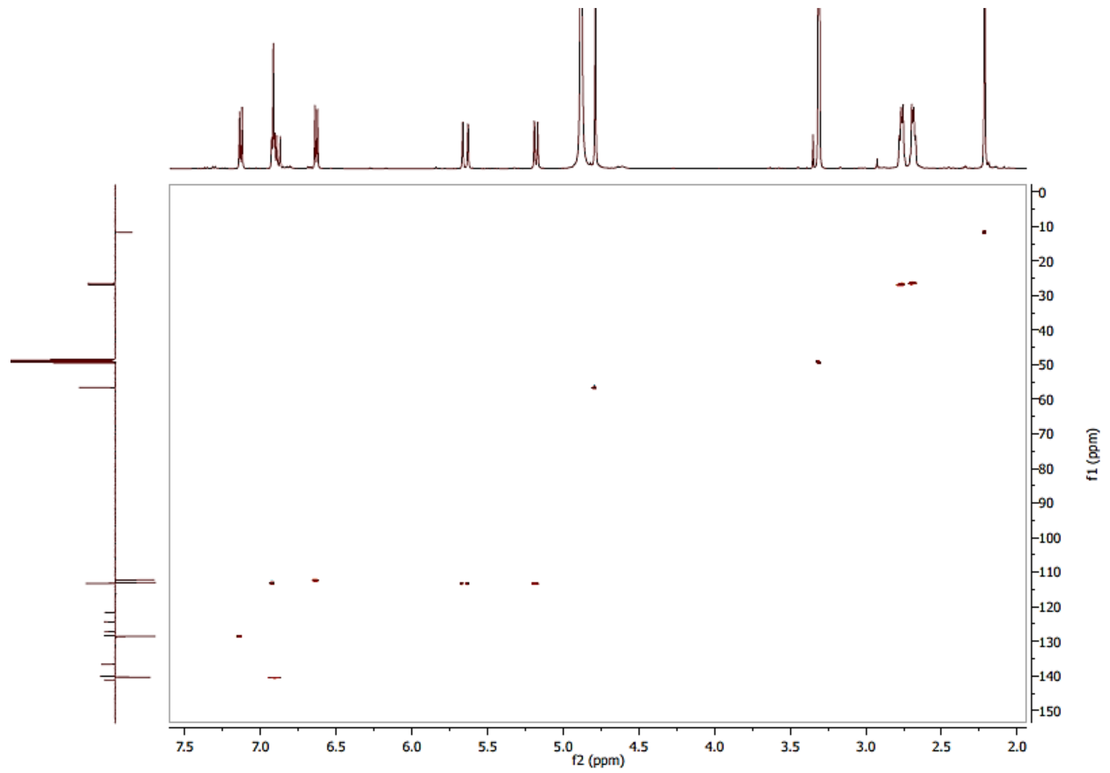

Figure S3. HSQC spectrum of maritin A (**1**) in methanol-d_4.

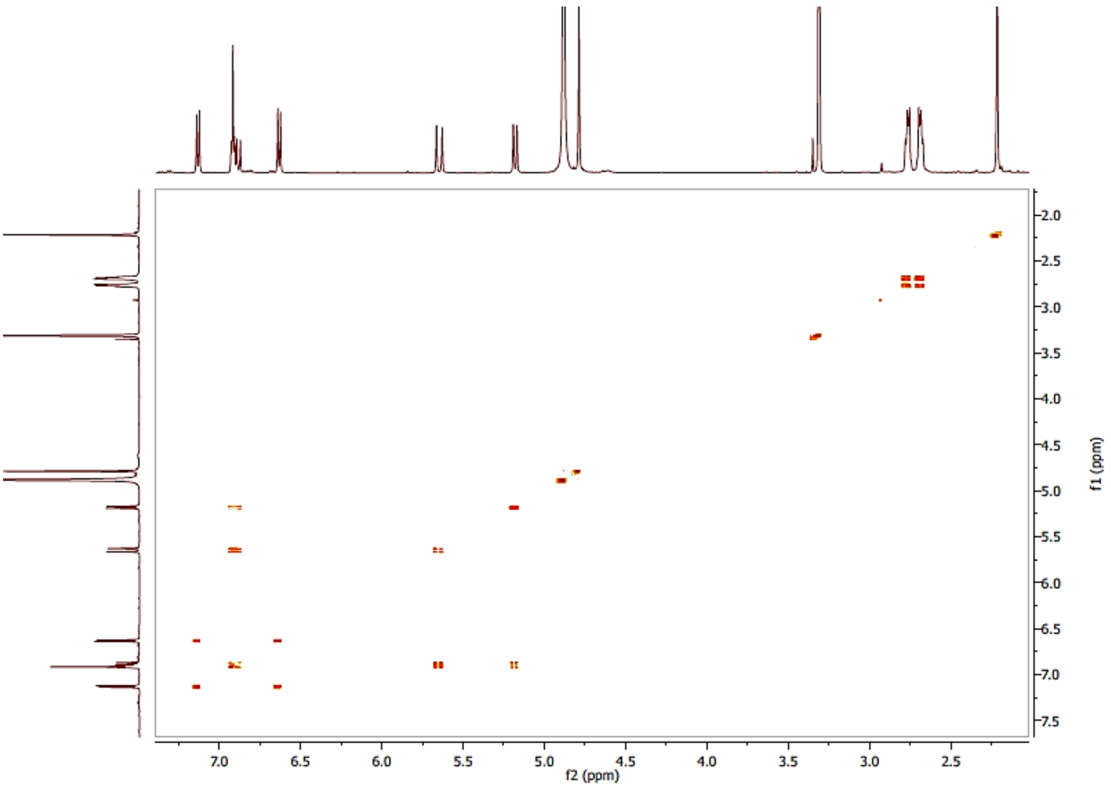

Figure S4. ^{1}H-^{1}H COSY spectrum of maritin A (**1**) in methanol-d_4.

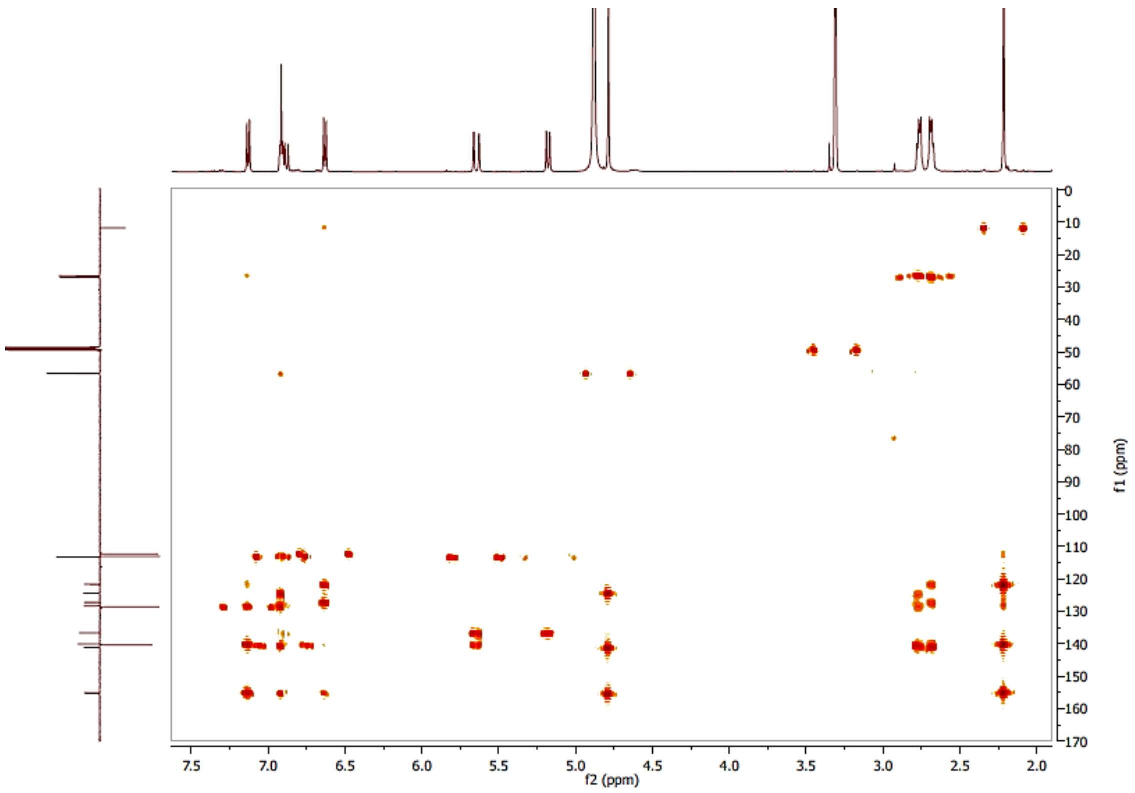

Figure S5. HMBC spectrum of maritin A (**1**) in methanol-d_4.

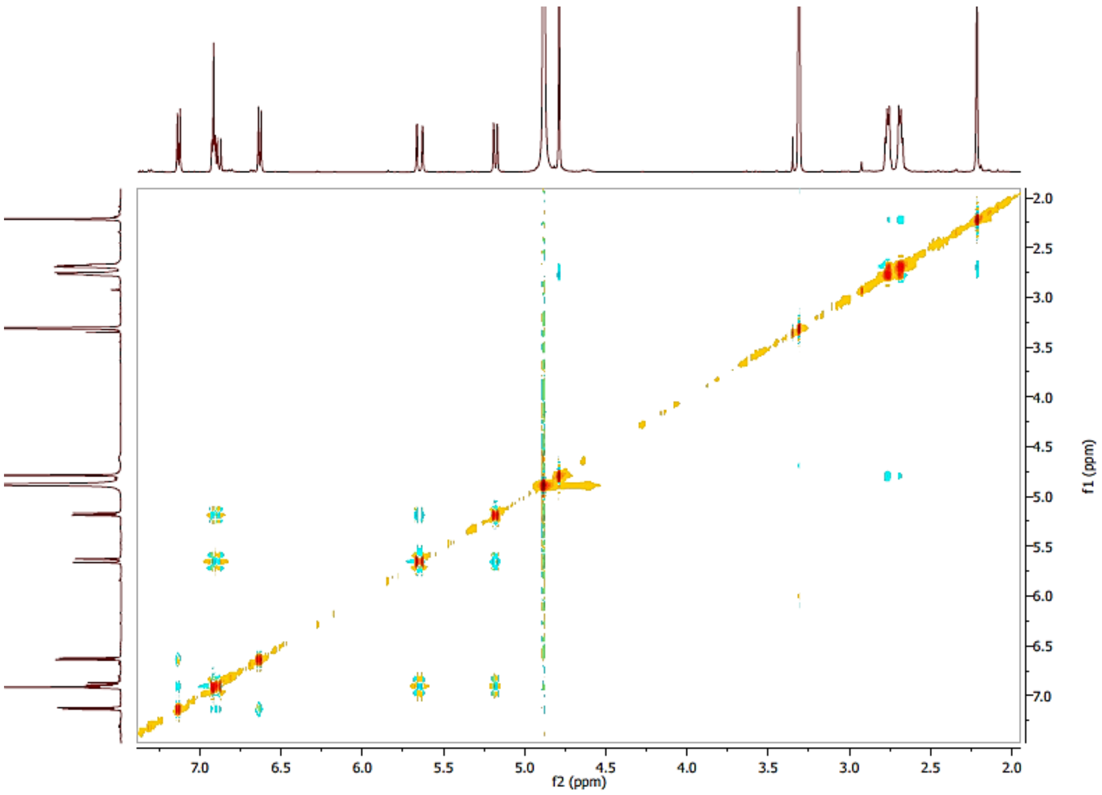

Figure S6 NOESY spectrum of maritin A (**1**) in methanol-d_4.

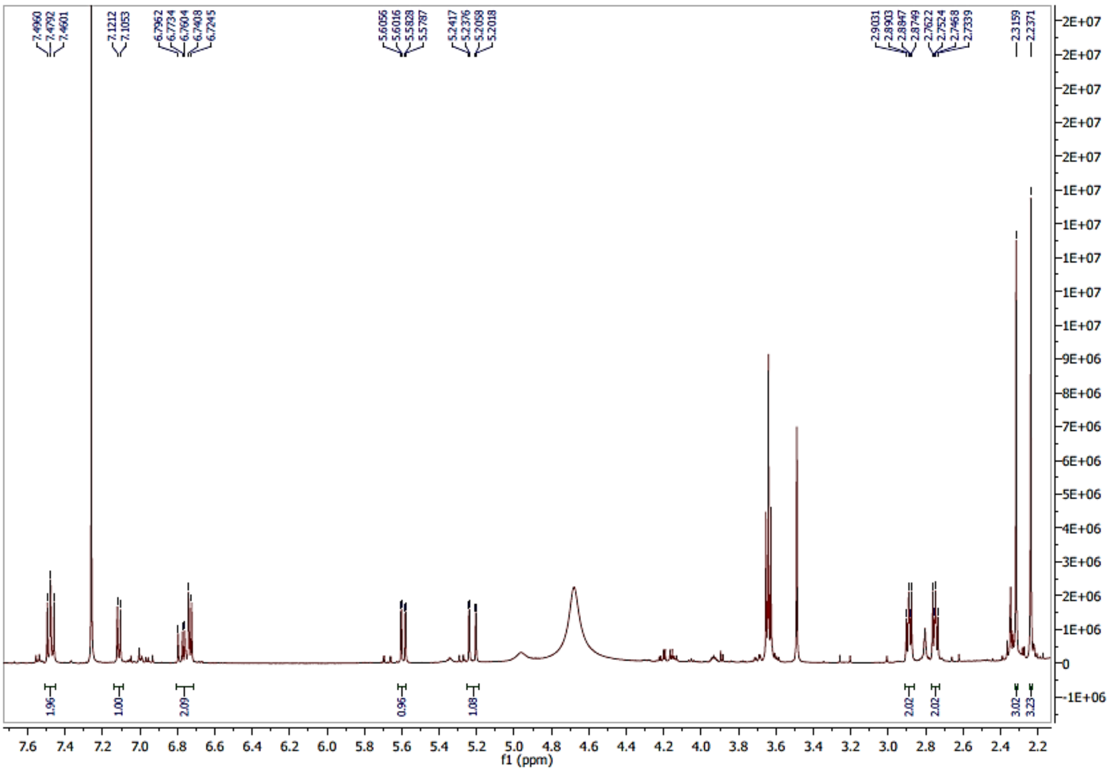

Figure S7. ^{1}H NMR spectrum (500 MHz) of maritin B (**2**) in $CDCl_3$.

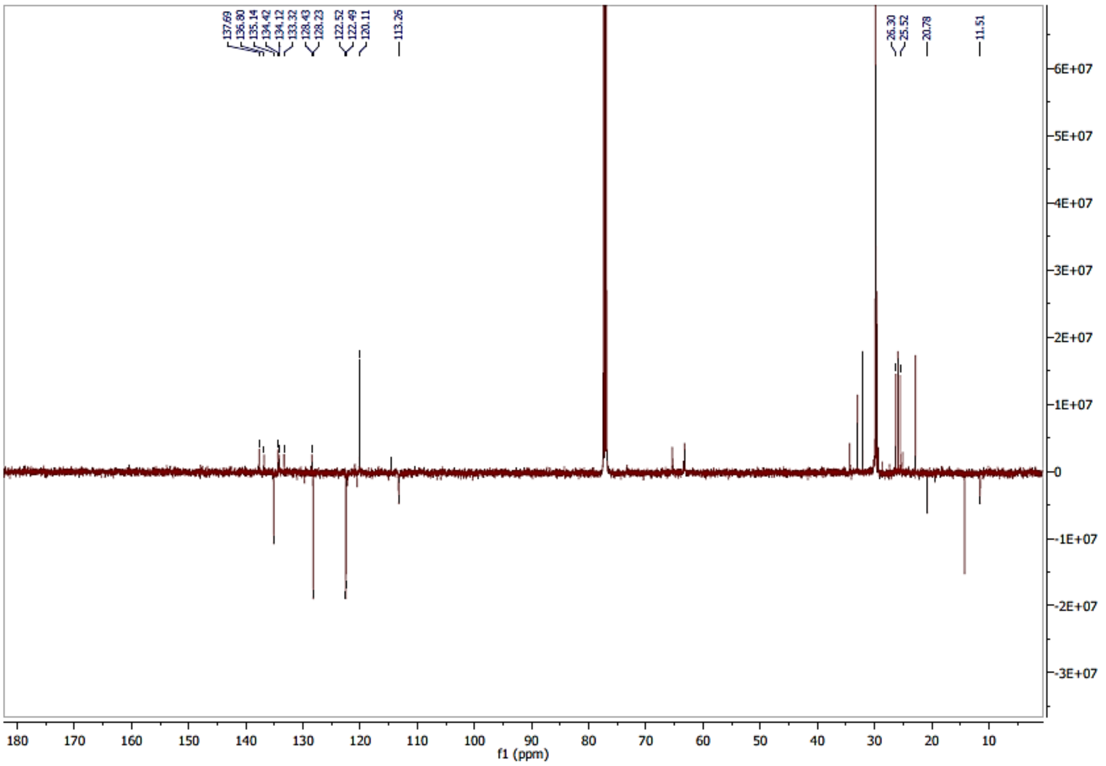

Figure S8. ^{13}C JMOD NMR spectrum (125 MHz) of maritin B (**2**) in $CDCl_3$.

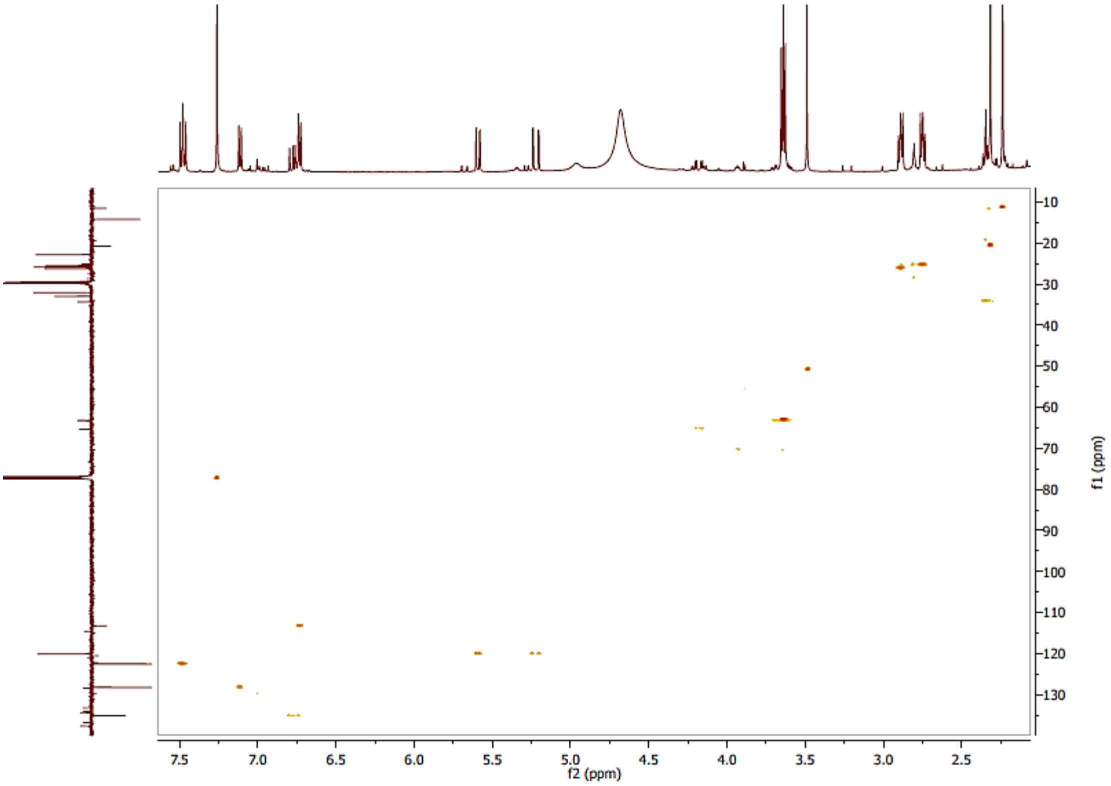

Figure S9. HSQC spectrum of maritin B (**2**) in $CDCl_3$.

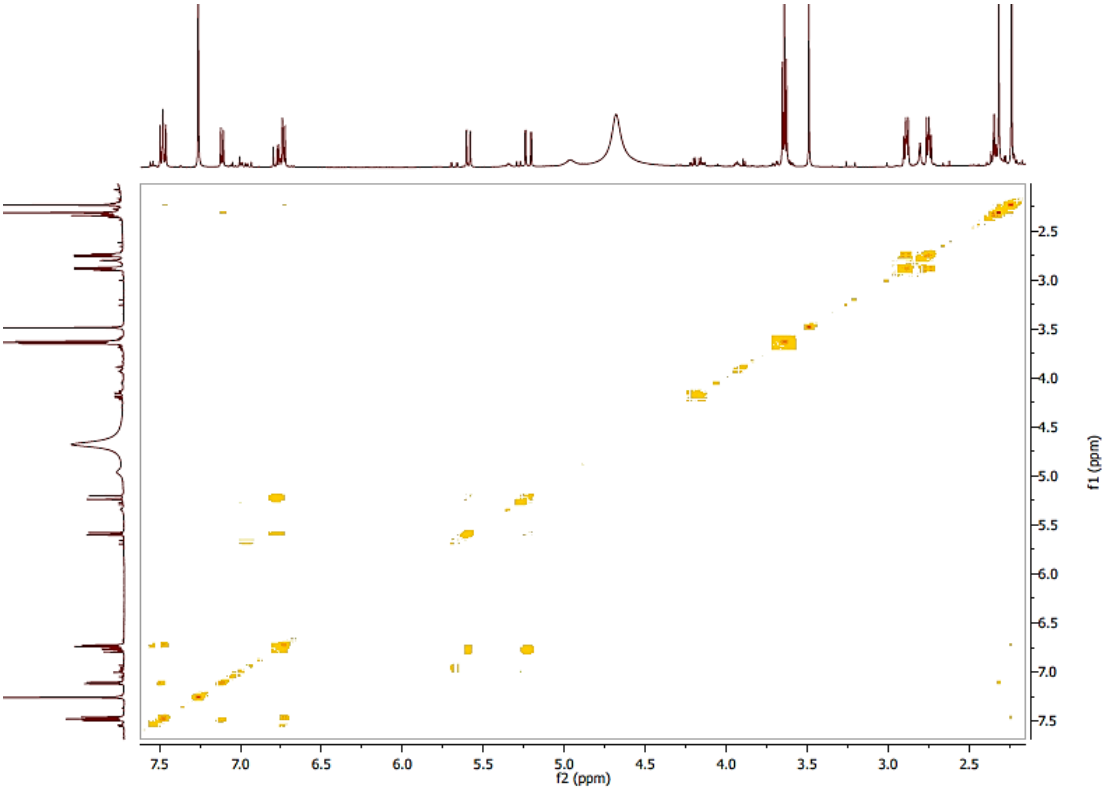

Figure S10. ^1H-^1H COSY spectrum of maritin B (**2**) in $CDCl_3$.

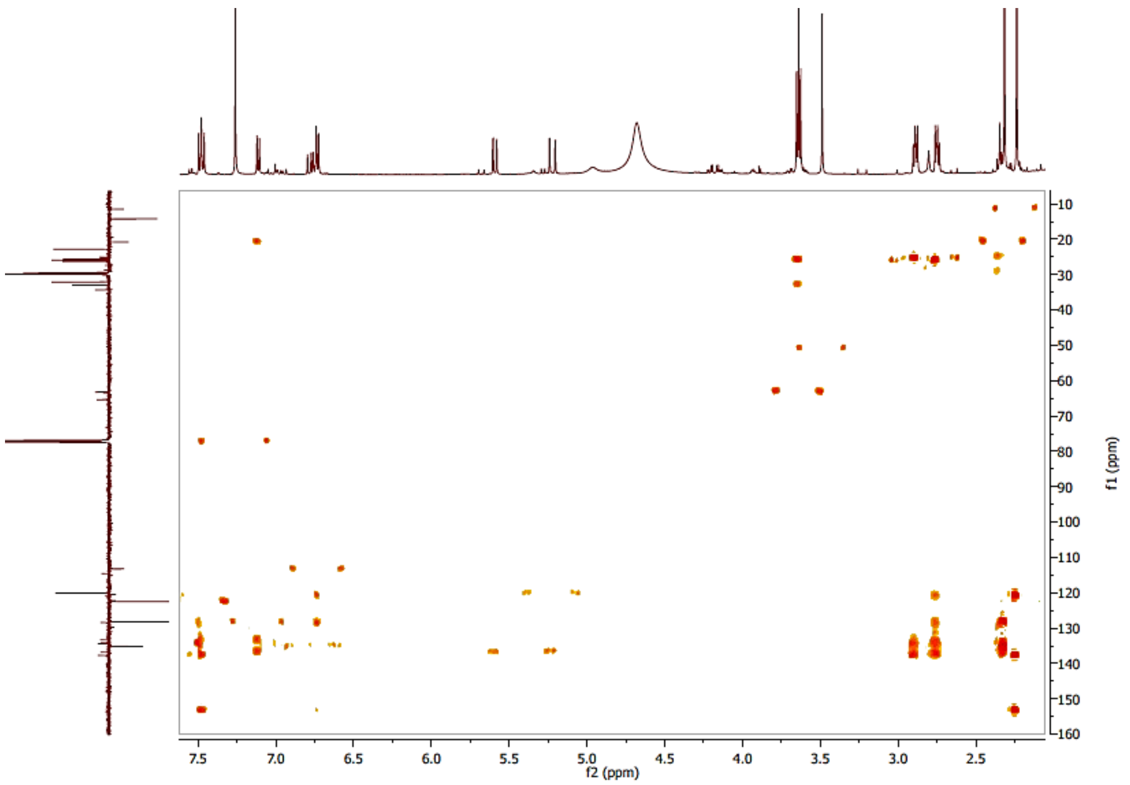

Figure S11. HMBC spectrum of maritin B (**2**) in $CDCl_3$.

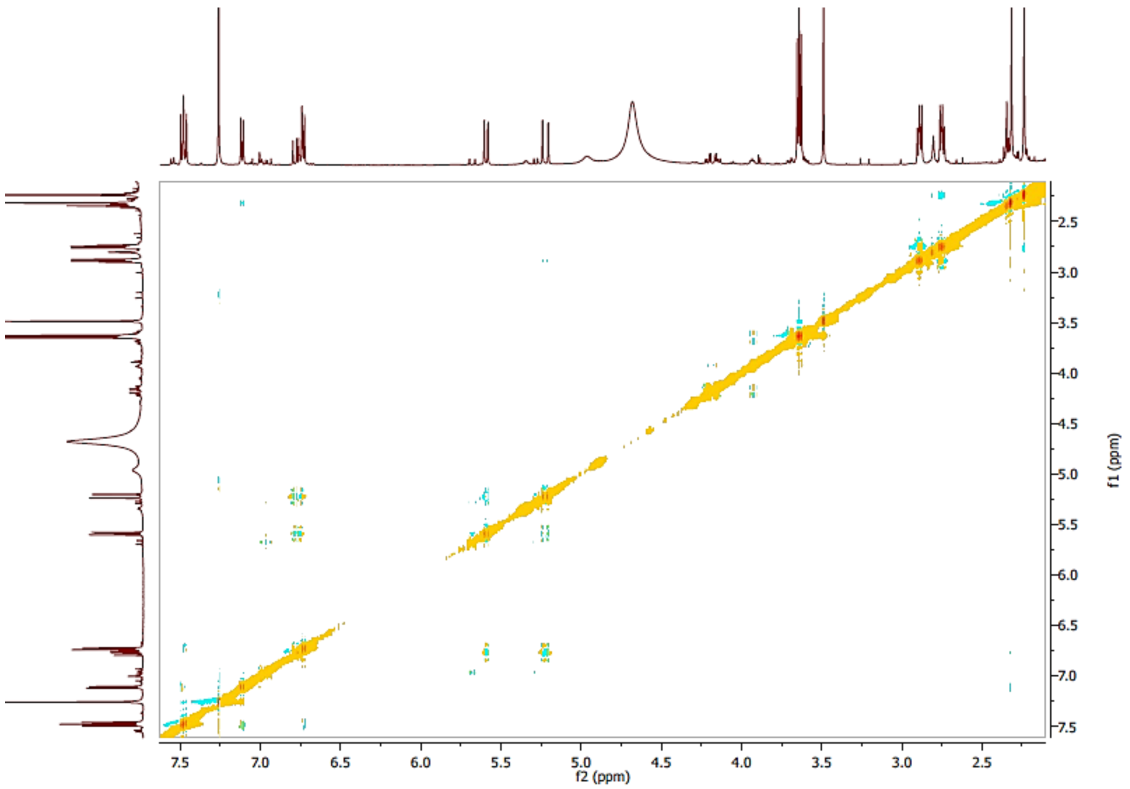

Figure S12. NOESY spectrum of maritin B (**2**) in $CDCl_3$.

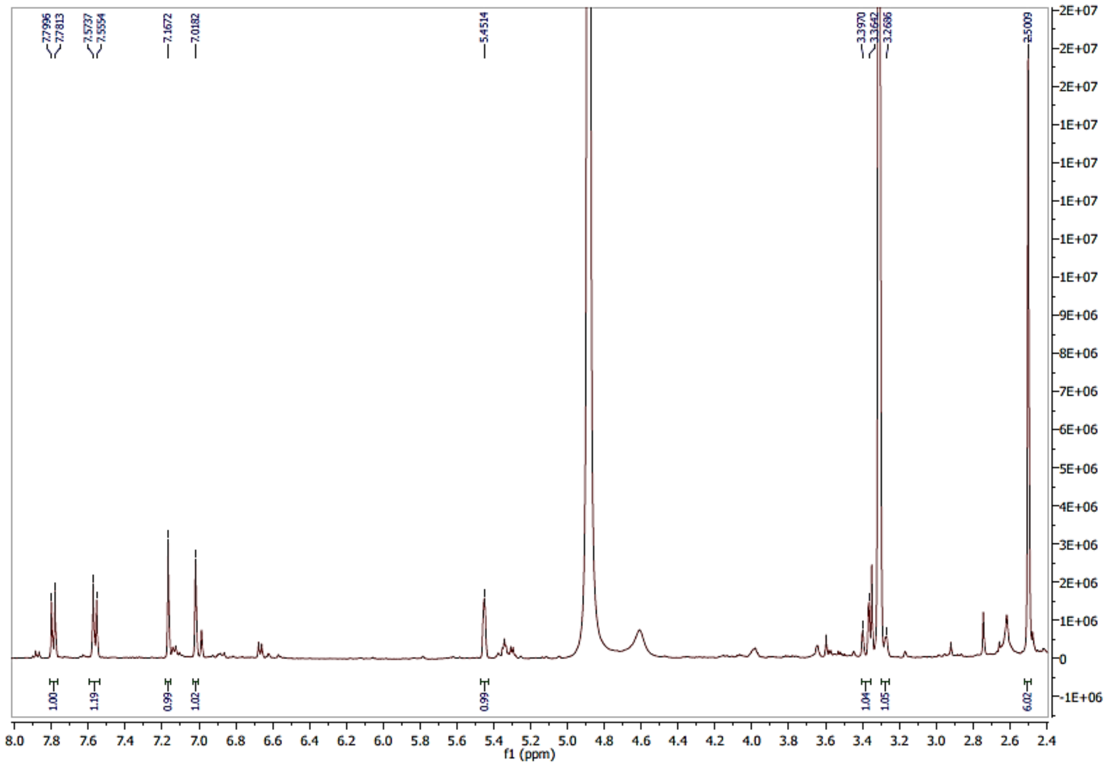

Figure S13. ^{1}H NMR spectrum (500 MHz) of maritin C (**3**) in methanol-d_4.

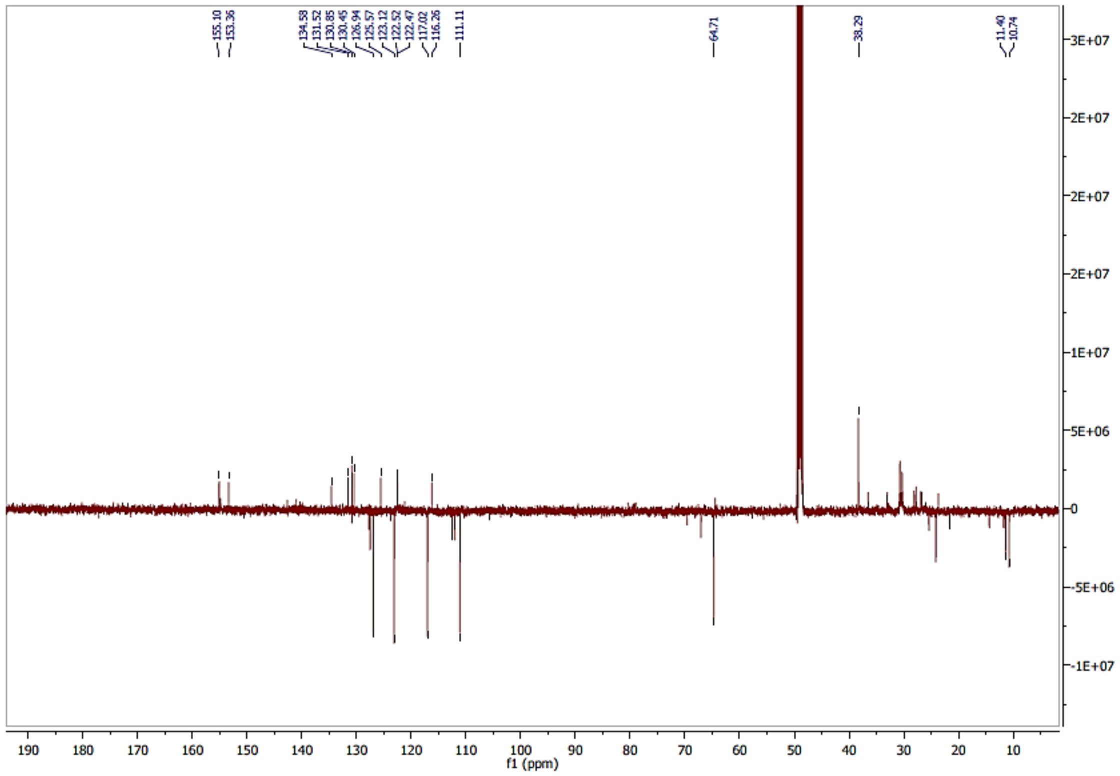

Figure S14. ^{13}C JMOD NMR spectrum (125 MHz) of maritin C (**3**) in methanol-d_4.

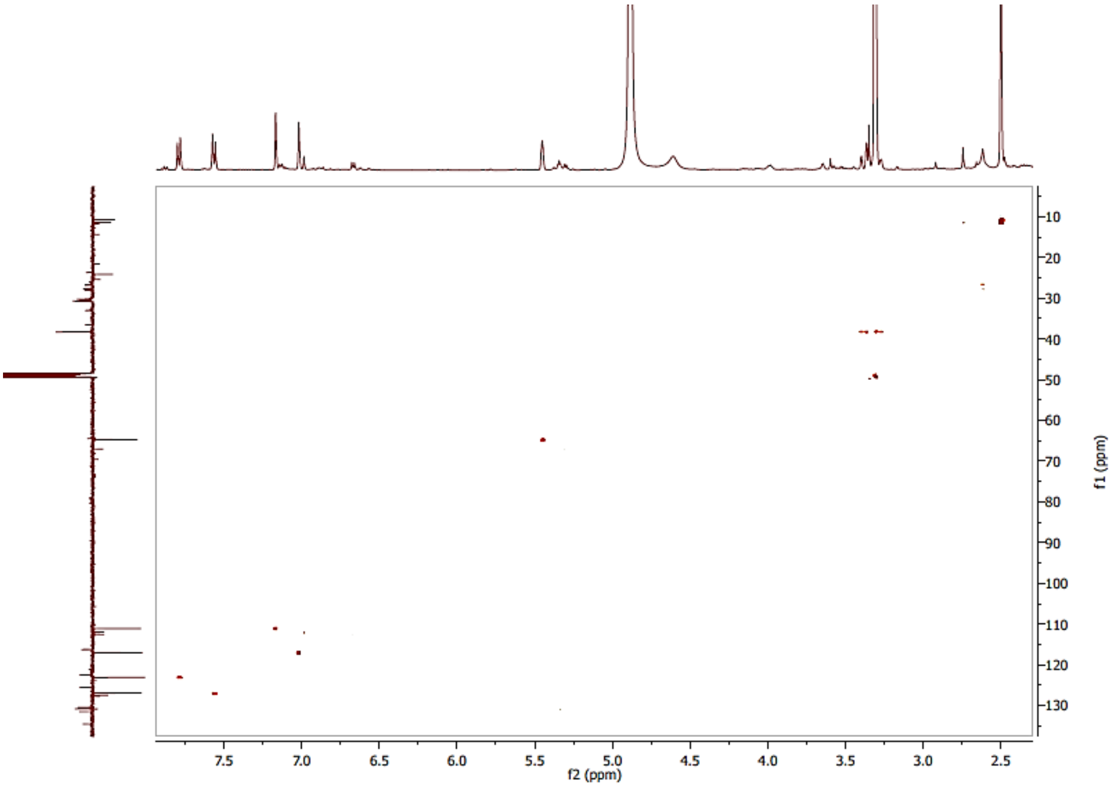

Figure S15. HSQC spectrum of maritin C (**3**) in methanol-d_4.

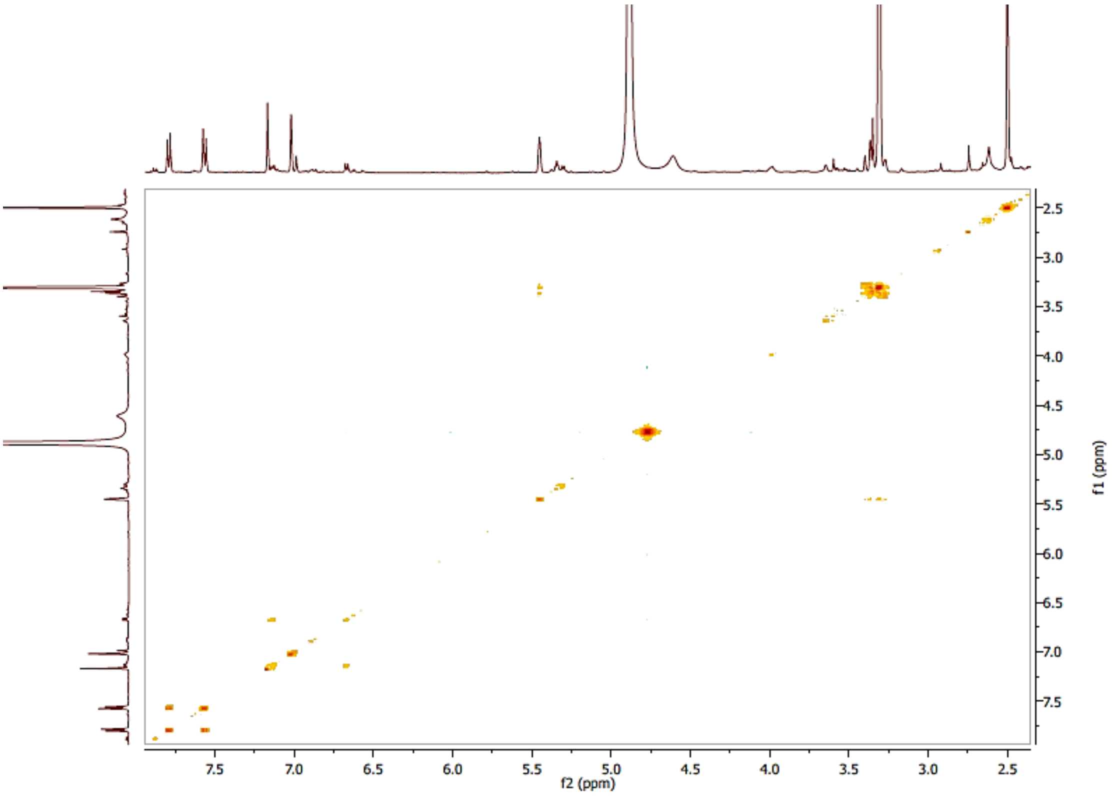

Figure S16. ^{1}H-^{1}H COSY spectrum of maritin C (**3**) in methanol-d_4.

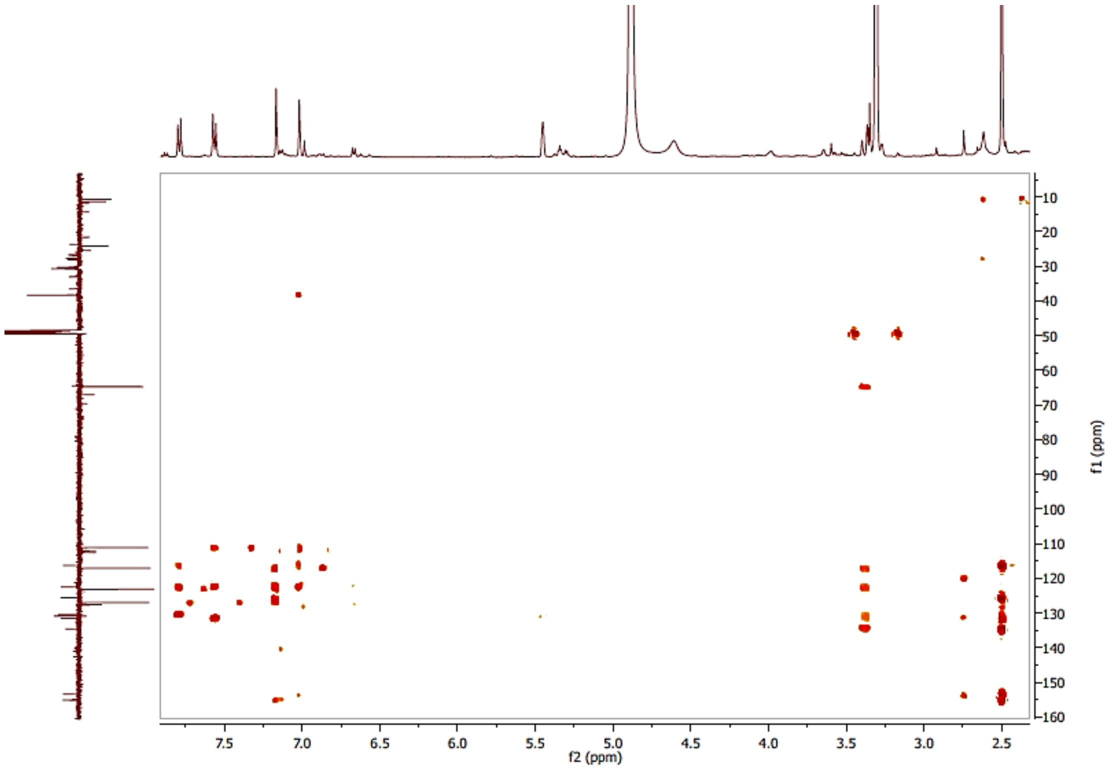

Figure S17. HMBC spectrum of maritin C (**3**) in methanol-d_4.

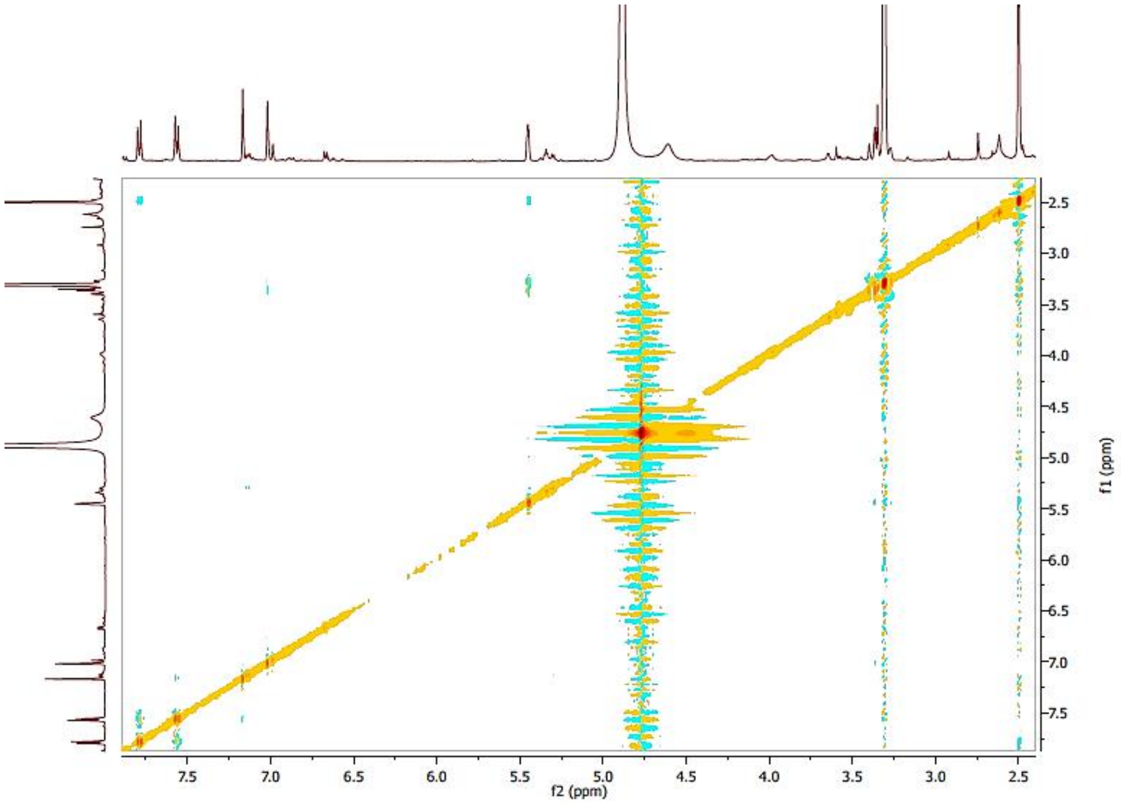

Figure S18. NOESY spectrum of maritin C (**3**) in methanol-d_4.

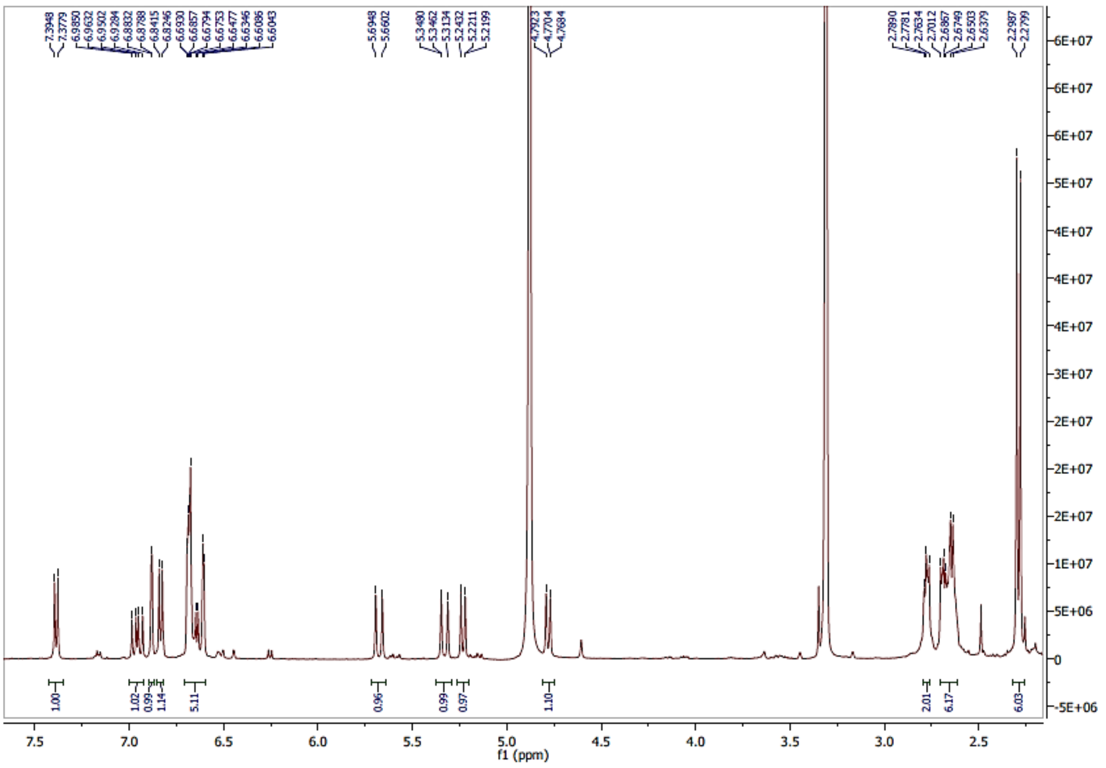

Figure S19. ^{1}H NMR spectrum (500 MHz) of maritin D (**4**) in methanol-d_4.

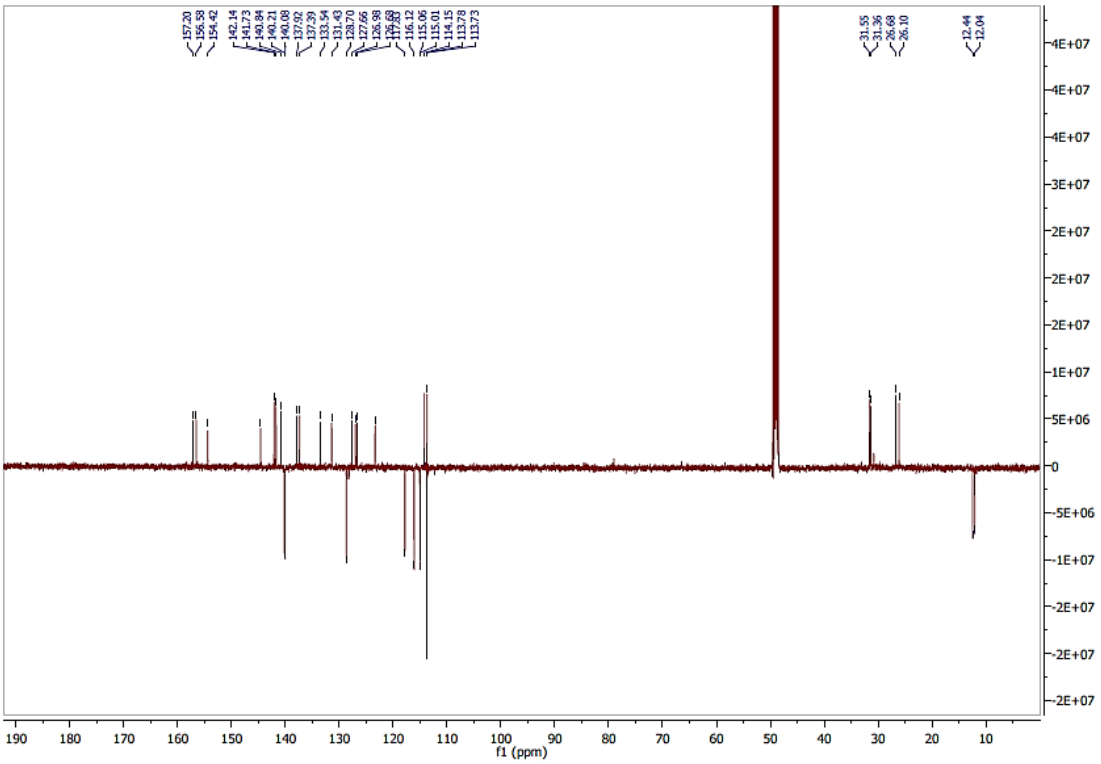

Figure S20. ^{13}C JMOD NMR spectrum (125 MHz) of maritin D (**4**) in methanol-d_4.

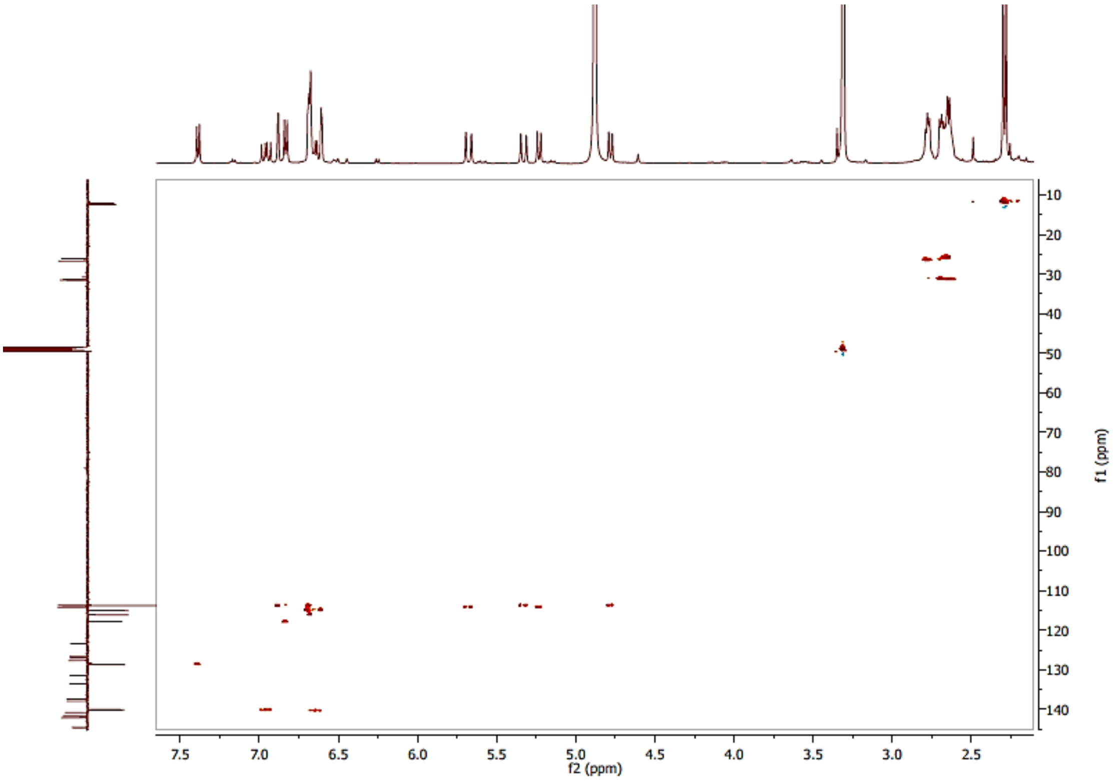

Figure S21. HSQC spectrum of maritin D (**4**) in methanol-d_4.

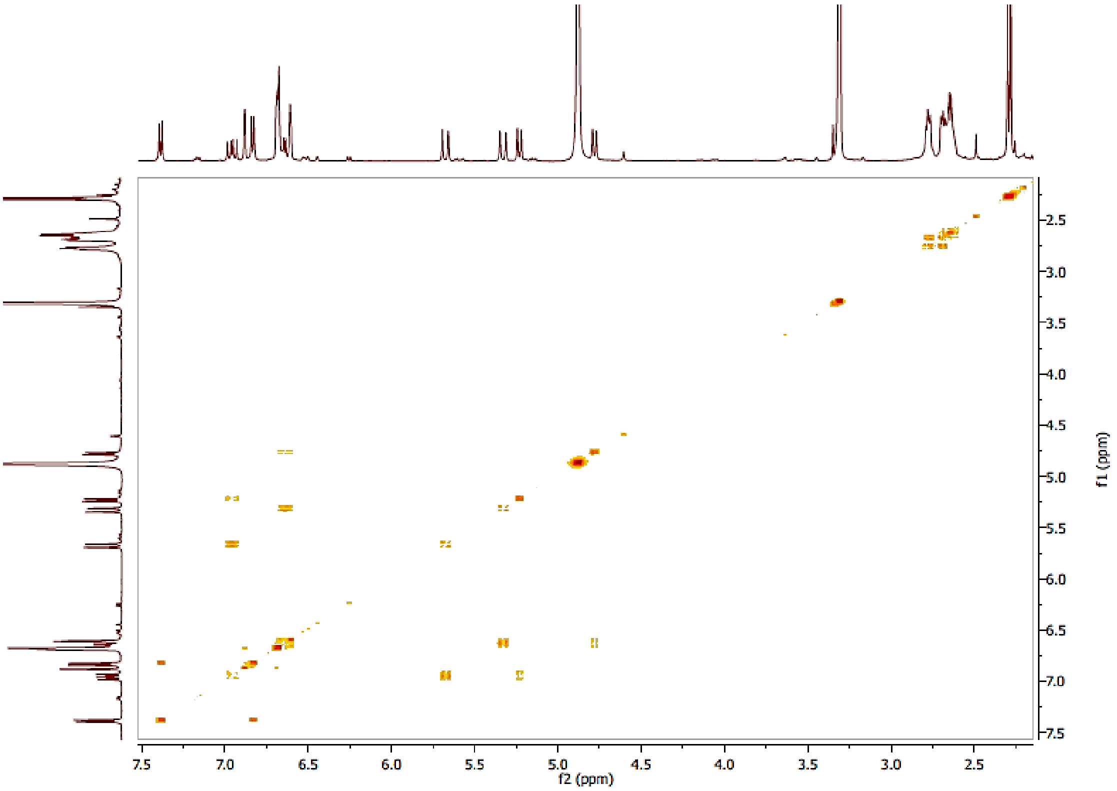

Figure S22. ^{1}H-^{1}H COSY spectrum of maritin D (**4**) in methanol-d_4.

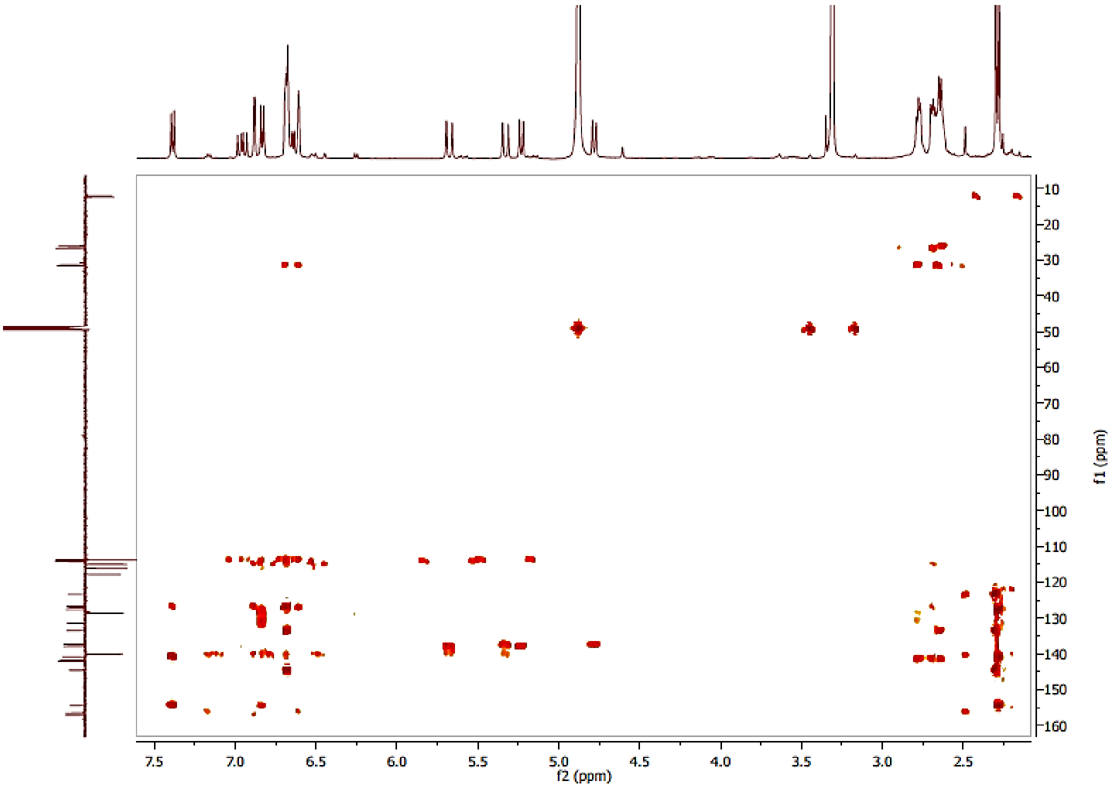

Figure S23. HMBC spectrum of maritin D (**4**) in methanol-d_4.

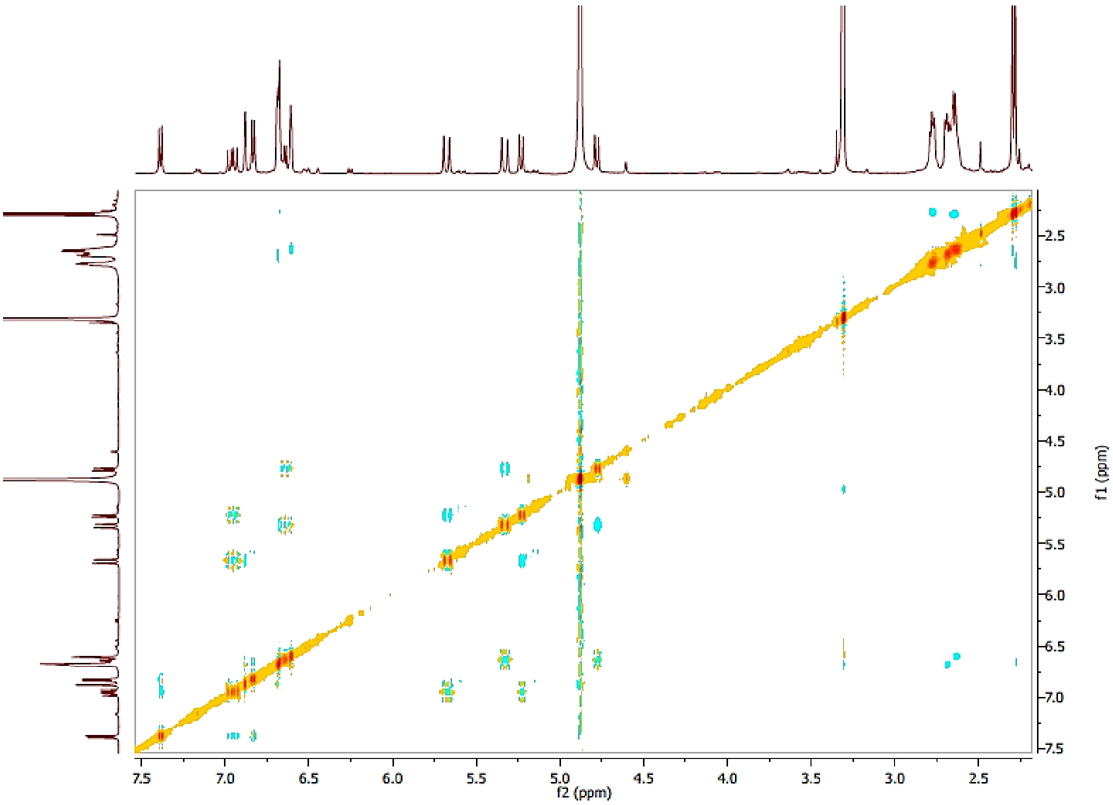

Figure S24. NOESY spectrum of maritin D (**4**) in methanol-d_4.

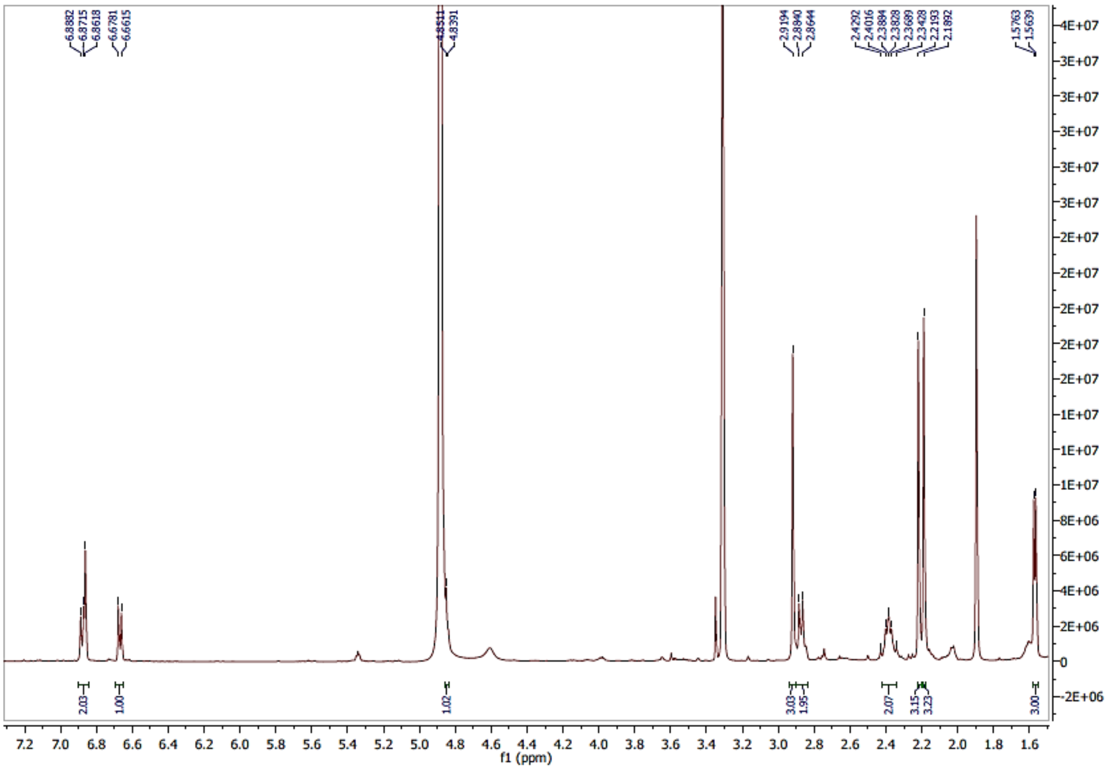

Figure S25. ^{1}H NMR spectrum (500 MHz) of jinflexin A (**10**) in methanol-d_4.

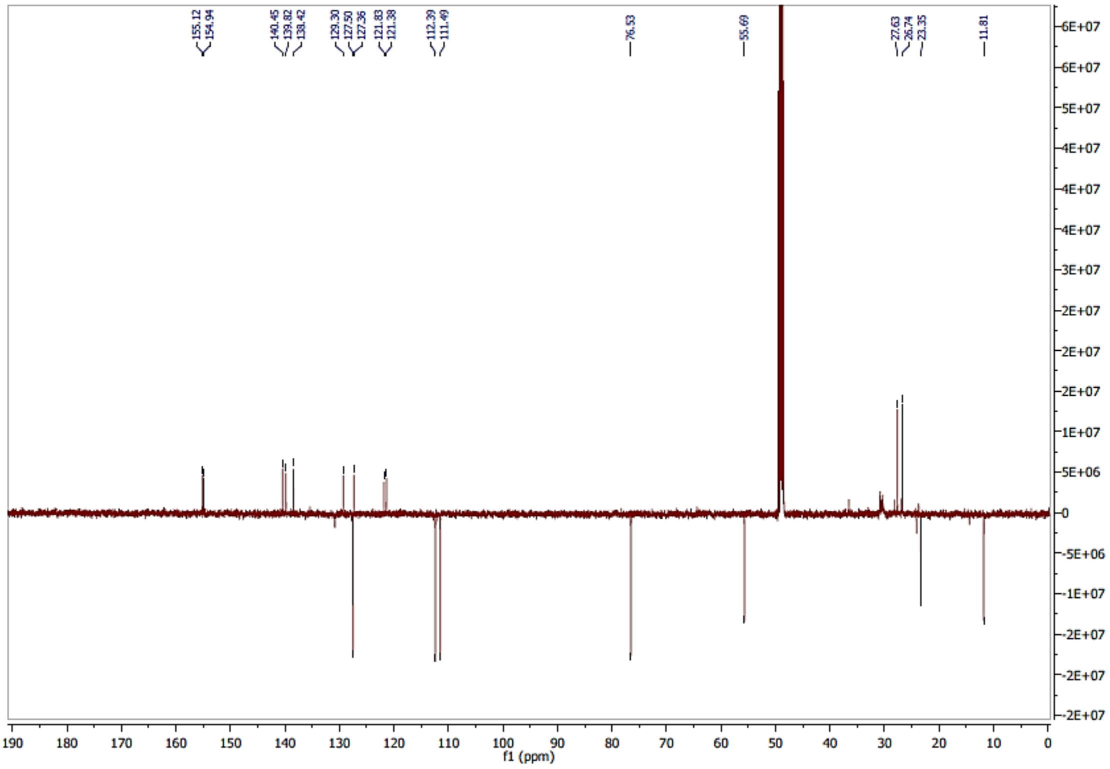

Figure S26. ^{13}C JMOD NMR spectrum (125 MHz) of jinflexin A (**10**) in methanol-d_4.
